# Identified RP2 as a prognostic biomarker for glioma, facilitating glioma pathogenesis mainly via regulating tumor immunity

**DOI:** 10.18632/aging.204962

**Published:** 2023-08-18

**Authors:** Yiyang Gong, Yun Ke, Zichuan Yu, Jingying Pan, Xuanrui Zhou, Yike Jiang, Minqin Zhou, Hong Zeng, Xitong Geng, Guowen Hu

**Affiliations:** 1 Department of Neurosurgery, The Second Affiliated Hospital of Nanchang University, Nanchang, Jiangxi 330006, China; 2 Second College of Clinical Medicine, Nanchang University, Nanchang, Jiangxi 330047, China

**Keywords:** RP2, biomarker, glioma, immune infiltration, independent prognostic factor

## Abstract

Glioma is the most common primary intracranial tumor in the central nervous system, with a high degree of malignancy and poor prognosis, easy to recur, difficult to cure. The mutation of Retinitis Pigmentosa 2 (RP2) can cause retinitis pigmentosa, it is a prognostic factor of osteosarcoma, however, its role in glioma remains unclear. Based on the data from TCGA and GTEx, we identified RP2 as the most related gene for glioma by WGCNA, and used a series of bioinformatics analyses including LinkedOmics, GSCA, CTD, and so on, to explore the expression of RP2 in glioma and the biological functions it is involved in. The results showed that RP2 was highly expressed in glioma, and its overexpression could lead to poor prognosis. In addition, the results of enrichment analysis showed that RP2 was highly correlated with cell proliferation and immune response. And then, we found significant enrichment of Macrophages among immune cells. Furthermore, our experiments have confirmed that Macrophages can promote the development of glioma by secreting or influencing the secretion of some cytokines. Moreover, we investigated the influence of RP2 on the immunotherapy of glioma and the role of m6A modification in the influence of RP2 on glioma. Ultimately, we determined that RP2 is an independent prognostic factor that is mainly closely related to immune for glioma.

## INTRODUCTION

Nearly 80% of primary malignant brain tumors are gliomas [1]. Glioma is divided into four types by the World Health Organization, with glioblastoma (WHO IV) accounting for 56.6% of all cases and possessing poor clinical prognosis, with only 41.4% relative survival rates in 1-year and 5.4% in 5-years, respectively. There are no specific diagnostic tools for glioma, which are mainly based on CT and MRI, and the final pathological diagnosis is made by tumour resection and biopsy. The treatment is mainly surgical, combined with radiotherapy and chemotherapy, but the treatment is usually not effective and is prone to recurrence, with a short prognosis for survival [2]. Furthermore, individuals using Immune Checkpoint Inhibitors (ICIs) have varying therapeutic efficacy, and in some circumstances, a poor therapeutic respondent has hampered their practical application [3]. Recent, Prognostic and predictive markers are crucial in medical practice for determining prognosis and selecting suitable therapy. That’s also especially significant in gliomas because of their potential complexity and diversity as well as the possibility of so-called pseudoprogression in MRI [4]. As a result, it is critical to identify effective molecular targets that might benefit to tailored treatment and better prognosis for glioma patients.

RP2 (retinitis pigmentosa 2) is a protein-coding gene consisting of five exons encoding a 350 amino acid predicted protein whose related pathways include ciliopathy and organelle biogenesis and maintenance [5]. RP2 is associated with GTP binding and GTPase activator activity [6]. RP2 has been reported to activate ARL3, which in turn leads to severe X-linked retinitis pigmentosa [7]. In addition, RP2 plays a role in tumorigenesis. RP2 has been proved to be significantly associated with immune infiltration of KIRC [6], which in turn affects the progression of KIRC. However, the role of RP2 in glioma is unclear.

The TCGA and GTEx are the two primary sources of the research data used in this work. In this study, we screened RP2 through a series of bioinformatics analyses dominated by WGCNA, examined the association between clinicopathological characteristics and RP2 mRNA expression in glioma. What’s more, the correlation between RP2 expression level and glioma prognosis was evaluated. In addition, we have investigated the mechanism of RP2 high expression in glioma and the relationship with the cell cycle and tumour-infiltrating immune cells. We also investigated the correlation between RP2 and Cytokines, TMB, as well as the impact of high RP2 expression on glioma immunotherapy. This study’s significance and uniqueness stem from the discovery of RP2 as a new, glioma-independent predictive factor that is intimately connected to cell proliferation and immune infiltration.

## MATERIALS AND METHODS

### Data sourcing and processing

Glioma mRNA expression data and clinical samples was collected from the TCGA Database (https://portal.gdc.cancer.gov/) and GTEx Database (https://commonfund.nih.gov) [8]. This study comprised 1158 normal samples and 706 GBMLGG samples for the gene expression profile. Clinical data from 599 GBM patients and 516 LGG patients were collected.

### Cell culture

Glioma U251 cell line was purchased from the Chinese Academy of Science (Shanghai, China). U251 cells were cultured in RPMI-1640 medium, and other cell lines were cultured in DMEM supplemented with 10% FBS and 1% penicillin-streptomycin. All cells were incubated in a 37°C incubator with 5% CO2.

### Weighted gene co-expression network analysis

WGCNA is an R package that can be used to find clusters of highly correlated genes [9], and the optimal soft threshold β was determined in order to achieve the condition of a scale-free network. In addition, modules were identified using a dynamic tree cutting method. Correlations between Eigengenes and clinical traits were analyzed to determine the modules that were significantly correlated with clinical traits. Gene connectivity was measured by the absolute value of Pearson correlation. Genes with high within-module connectivity were identified as the central genes of the module.

### TIMER database analysis

The TIMER (https://cistrome.shinyapps.io/timer/) is a collection of tools for systematically analyzing immune infiltrations in various cancer types [10]. The “Gene” and “scna” modules were used to predict the correlation among the infiltration of immune cells in each tumor sample and RP2 mRNA expression and copy number variation data in GBMLGG. And analyse the relationship between immune cell gene markers and other important contents with the help of the “Correlation” module. We also investigated the relationship between RP2 in pan-cancer and different immune cell infiltration.

### SMART

SMART (Shiny Methylation Analysis Resource Tool) (http://www.bioinfo-zs.com/smartapp), an easy-to-use website based on data from TCGA, focuses on the correlation analysis of DNA methylation [11]. We used it to achieve CpG visualization of RP2 on the chromosomes.

### MethSurv

MethSurv (https://biit.cs.ut.ee/methsurv/) is a web application that uses DNA methylation data to do variable survival analysis. The prognostic value of hypomethylation at some CpG sites in LGG and GBM were explored.

### LinkedOmics

LinkedOmics (http://www.linkedomics.org/login.php), an online analysis site on the basis of TCGA tumor samples, designed to analyze multidimensional data of 32 kinds of tumors [12]. Using the “LinkFinder module”, the co-expressed genes linked to RP2 in the TCGA-GBMLGG database were visualized with volcano plots and heat maps. In addition, the effect of RP2 mRNA high expression on prognosis of glioma patients was analyzed in the same module. What’s more, we made Gene Ontology (GO) and Kyoto Encyclopedia of Genes and Genomes (KEGG) analysis in “LinkInterpreter module”, to seek functional enrichment of co-expressed genes mentioned above. Pathways with FDR <0.05 were considered as meaningful.

### GlioVis

The GlioVis (http://gliovis.bioinfo.cnio.es/) is an online platform used by us for data visualization and analysis to explore a wide range of information on the clinicopathological features and prognosis of gliomas [13].

### Gene set enrichment analysis

GESA is a method for interpreting gene expression data, embodied in a software package together with a database of 1,325 gene sets. Relevant RNA-seq data of GBMLGG were downloaded from Genomic Data Commons (https://portal.gdc.cancer.gov/) [14]. The samples were divided into two groups according to the expression of RP2, and using this method we analyzed the enrichment of gene set in which RP2 is located. The parameters were established such as: gene set database: h. All. V7.4 Symbols. gmt (Hallmarks); number of permutations: 1,000. *P* value < 0.05 and FDR < 0.05 were considered as meaningful.

### PPI network construction

STRING (https://string-db.org/) is usually adopted for exploring physical interactions or functional associations between proteins. With the help of it, we built a Protein-Protein Interaction Network (PPI) with the top 500 genes most closely related to RP2 from volcano plots. We studied the connection among these genes. The parameter of medium confidence was set at 0.9. Then, the top 500 genes were evaluated by Cytoscape 3.9.1, and a functional cluster analysis were performed with MCODE. The selection criteria are as follows: Max depth = 100, node score cutoff = 0.2, K-core = 2.

### GeneMANIA analysis

GeneMANIA (http://www.genemania.org) [15], is a truly powerful online resource for gene function and lists analyses. We used it to draw an interactive functional network of RP2, mainly to find proteins with strong physical interaction with RP2. In the network, we used lines in various thickness and colors to show the functional relationship and correlation strength between the two connected ends.

### cBioPortal analysis

The cBioPortal for Cancer Genomics (https://www.cbioportal.org/) is a resource for discovering, visualizing, and analyzing multidimensional genomics data [16]. “Mutations” module was utilized to found out RP2 and its physical interaction protein in glioma.

### Protein structure and docking analysis

RCSB Protein Data Bank (https://www.rcsb.org/) enables breakthroughs in exploration of 3D protein structure. The structure of Retinitis Pigmentosa 2 (RP2) was obtained from PDB ID: 2BX6; the structure of ARL3 was obtained from PDB ID: 4GOJ; the structure of WDR83 was obtained from PDB ID: Q9BRX9. The binding patterns between RP2 and ARL3 and between RP2 and WDR83 were studied by docking through the Z DOCK web server (https://zlab.umassmed.edu/zdock/index.shtml) and visualized, analyzed and plotted using PYMOL.

### TISIDB

TISIDB (http://cis.Hku.hk/TISIDB/) offers a wealth of information about tumor immunity and makes it possible to do thorough research on tumor-immune interactions [17]. There, we looked for a relationship between RP2 and six aspects of the immune system in gliomas (lymphocytes, immunomodulators, chemokines, etc.).

### GSCA analysis

GSCA (http://bioinfo.life.hust.edu.cn/GSCA) is an integrated platform for genomic, pharmacogenomics and immunogenomic genomic gene set cancer analysis [18]. Combining clinical information and small molecule drugs, users can mine candidate biomarkers and valuable drugs for better experimental design and further clinical trials. We used it to uncover the drug sensitivity of ARL3, SLC44A2, WDR83, RP2 and OSTF1.

### CTD

The Comparative Toxicogenomics Database (CTD, http://ctdbase.org/) is a digital resource that facilitates the study of novel links in the molecular mechanisms by which chemical substances affect health outcomes. We have used this database to query and visualize the interacting drugs or small molecules of RP2.

### Quantitative RT-PCR analysis

Total RNAs were extracted using the QIAGEN RNeasy mini kit, and reverse transcription reactions were performed using the ABI Taqman Reverse Transcription Reagents. After mixing the generated cDNA templates with primers/probes and ABI Taqman Fast Universal PCR Master Mix, reactions were performed with the ABI-7900 Fast Real-time PCR system and SYBR green qPCR Mastermix from Agilent Technologies Stratagene. This technique was adopted in our study to examine the relative mRNA expression of TGF-β and IL-10.

### Statistical analysis

R software (version 4.2.1) was used to conduct all statistical analysis in this research. The different expression of RP2 was detected by rank-sum test, meanwhile, the “limma” and “beeswarm” packages were driven. Multivariate Cox regression analysis screened factors significantly related to prognosis (*p* < 0.05) (Cox model uses the “survival” and “survminer” packages of “R”). SurvivalROC (version 1.0.3) was used to produce the ROC curve for evaluating the ability of RP2 expression level to predict 1-year, 3-year, or 5-year survival. We used the “Survival” and “SurvMiner” packages to produce the survival scene of RP2 in pan cancer. The R software package MAfTools (version 2.8.05) was used to calculate the TMB (Tumor mutation burden) of each Tumor by TMB function. The correlation between RP2 expression and immune checkpoint-related genes using R software (“pheatmap” packages). The particular status of the tumor mutation load was shown by log transformation after we combined the TMB and gene expression data of samples.

### Data availability statement

Publicly available datasets were analyzed in this study. The data are accessible in TCGA and GTEx databases. Further inquiries can be directed to the corresponding author.

## RESULTS

### Weighted gene coexpression network analysis was used to identify the key gene in glioma

**Figure 1:**
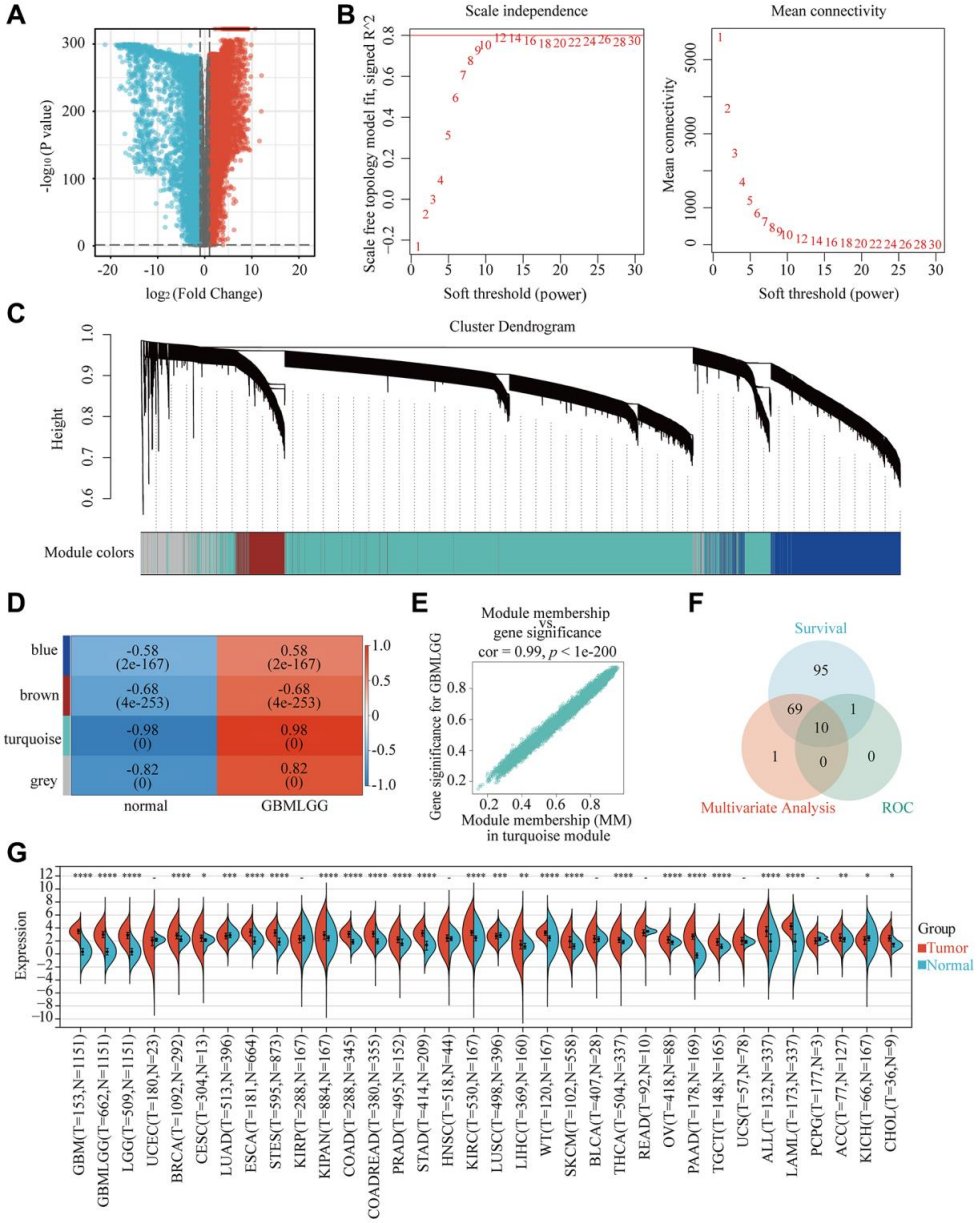
**Identification of the key gene modules in WGCNA.** (**A**) Volcano map showed differentially expressed genes. (**B**) Determination of the soft-thresholding power. (**C**) Dendrogram of differentially expressed genes clustered based on a dissimilarity measure (1-TOM). (**D**) The correlation of gene modules with clinical traits. (**E**) Gene correlation scatter plot of the turquoise module. (**F**) The Venn diagram showed WGCNA combined genetic features to screen out 10 genes. (**G**) The expression of RP2 in 34 kinds of cancers.

To identify pivotal genes in glioma, we first discovered that 8762 genes were upregulated, while 6964 were downregulated in glioma tissue (Figure 1A). Following that, β = 12 was selected as the soft threshold for implementing a scale-free network (Figure 1B). Using the dynamic tree cutting package, 7 modules were defined (Figure 1C). It should be noted that the correlation heatmap for module traits revealed that the turquoise module had the highest association with glioma (Figure 1D). The gene distribution results in the turquoise module displayed that glioma and Module membership (MM) were highly associated, suggested that genes in this module were highly significantly correlated with glioma (Figure 1E). Furthermore, Venn diagram showing 10 intersecting genes, which were most associated with glioma, screened by the WGCNA method combined with Multivariate Analysis, ROC and survival curves (Figure 1F). What’s more, RP2 expression was significantly higher in GBMLGG, founded in the TIMER database (Figure 1G). Finally, by inquiring background information, we selected RP2, which has not been reported in glioma and has the best results, as our research target.

### Association of RP2 expression and clinicopathological characteristics in glioma

**Figure 2:**
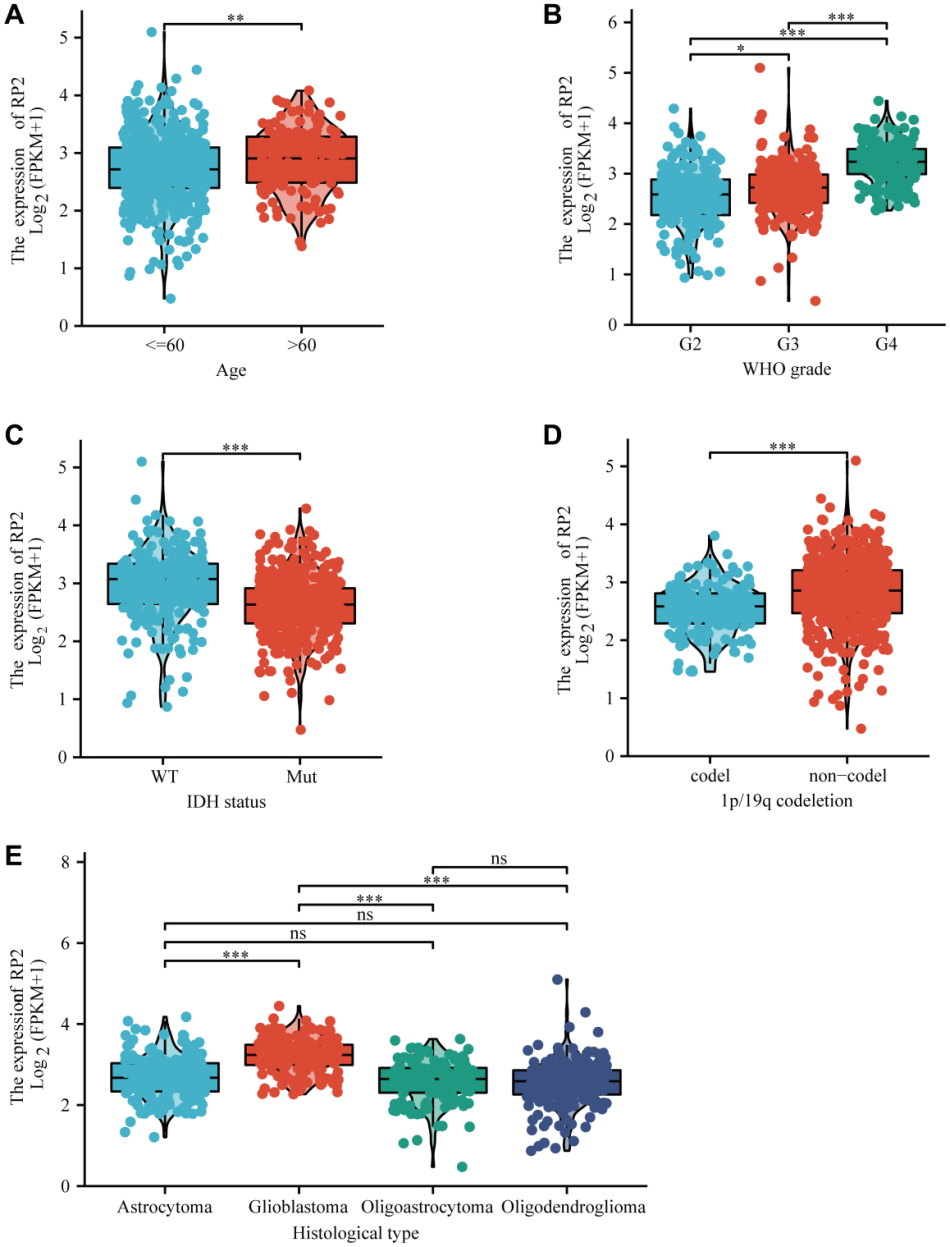
**The box plot showed the association of RP2 expression with clinicopathological characteristics.** (**A**) Age, (**B**) grade, (**C**) IDH status, (**D**) 1p/19q codeletion, (**E**) histological type.

In order to determine the relevance of RP2 expression to clinicopathological characteristics, we used R software to analyse. We noticed that RP2 mRNA expression was upregulated in patients above 60, with higher tumour grade, unmutated group of IDH and in the non-coding 1P/19Q (Figure 2A–2D). Futhermore, we discovered that the mRNA expression of RP2 was most pronounced in glioblastoma among all histological types (Figure 2E). These outcomes conferred that the RP2 expression was intimately associated with the clinicopathological characteristics.

### Over-expression of RP2 was associated with awful prognosis of glioma patients

**Figure 3:**
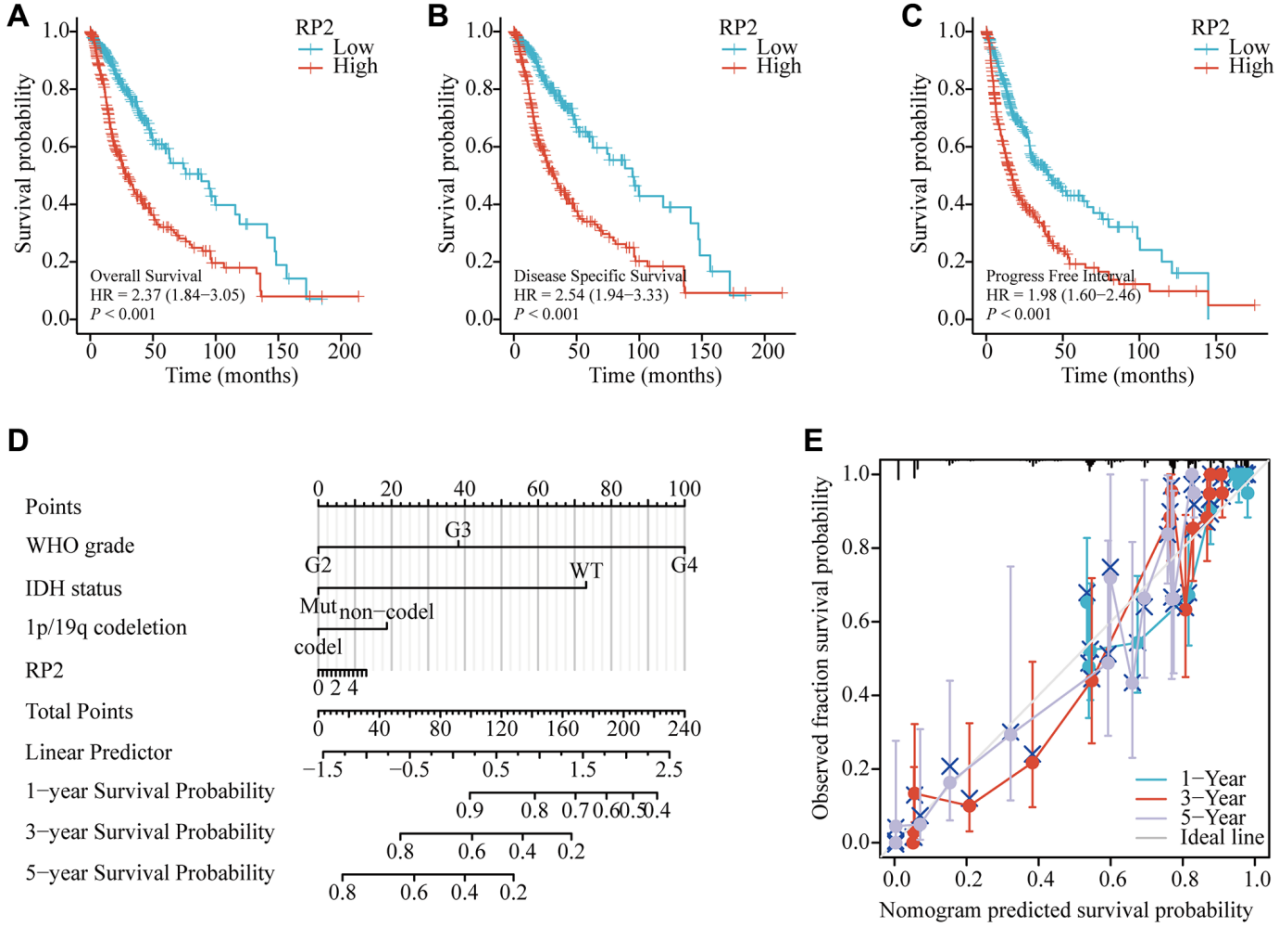
**Relationship between RP2 and prognosis of glioma patients.** Glioma patients with lower expression level of RP2 had favorable (**A**) OS (HR = 2.37, *p* < 0.001), (**B**) DSS (HR = 2.54, *p* < 0.001), (**C**) PFS (HR = 1.98, *p* < 0.001). (**D**) Nomograms and (**E**) calibration curves.

To further find the underlying mechanism of RP2 over-expression in glioma patients, we analyzed the effects of RP2 over-expression in TCGA databases on overall survival (OS), disease-free survival (DSS), and progression-free survival (PFS) of glioma patients and plotted survival curves. The findings showed that patients with high RP2 expression had proportionately lower OS, DSS, and PFS times than the low expression group, demonstrating that high RP2 expression was correlated with worse prognosis (Figure 3A–3C). Overall survival was also examined using LinkedOmics to further demonstrate the link between RP2 expression and glioma patient prognosis (Supplementary Figure 1A), and the high and low RP2 expression groups were differentiated by the GlioVis in another way, and both results indicated that the high RP2 expression group had a worse survival expectation than the low RP2 expression group (Supplementary Figure 1B–1D). Besides, in order to forecast the overall survival of glioma patients in the TCGA cohort, we developed a nomogram. The WHO grade for the malignancy, IDH status, 1p/19q codeletion, and RP2 were included in the nomogram as prognostic variables. (Figure 3D). And the calibration curve displayed that the nomogram was credible in predicting possibility of 1-, 3-, 5-years overall survival in glioma (Figure 3E). In conclusion, our data suggested that a worse prognosis for glioma patients is frequently linked to increased RP2 expression.

### Correlation between RP2 hypomethylation and prognosis in LGG and GBM

**Figure 4:**
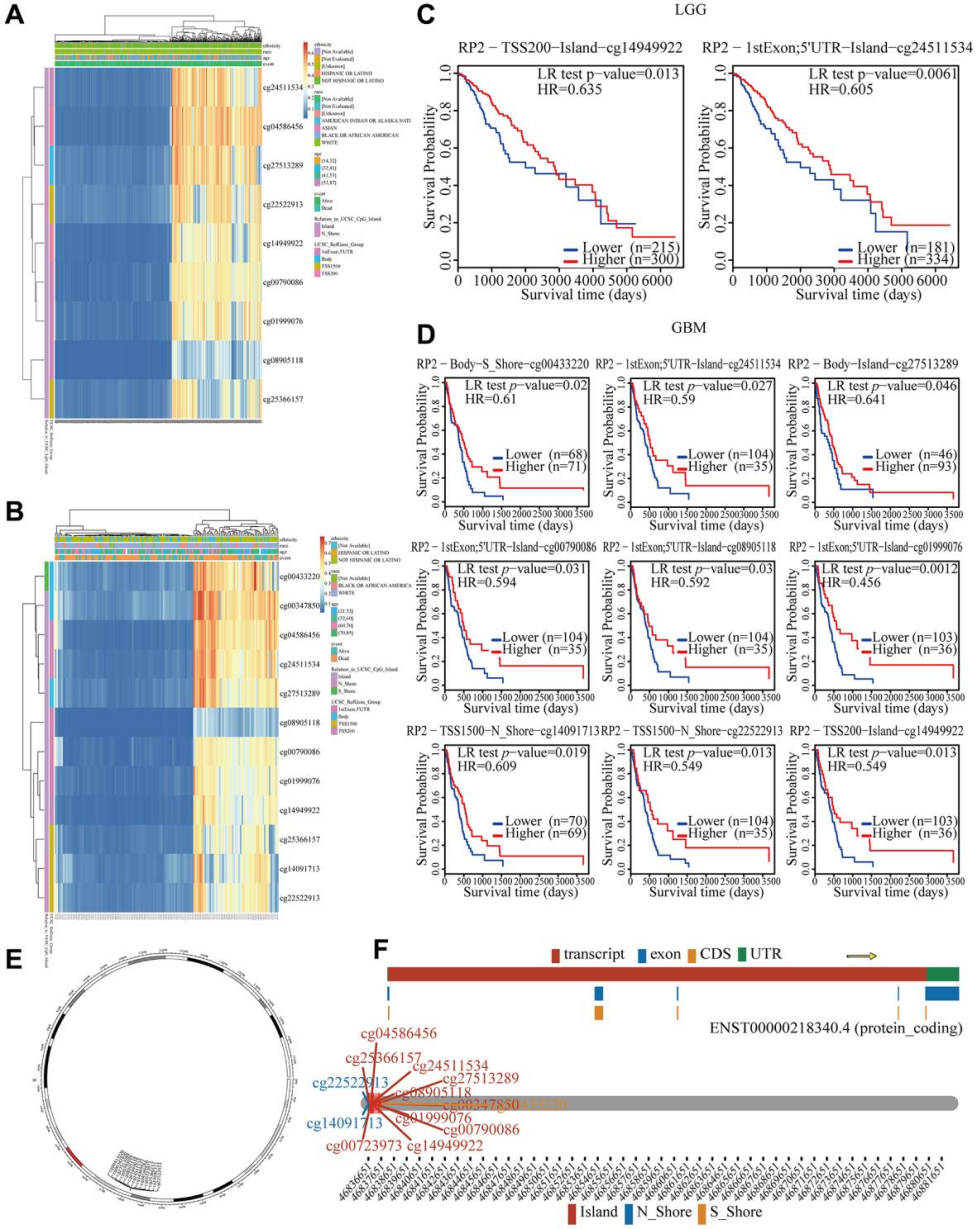
**Correlation between RP2 hypomethylation, and prognosis in GBM and LGG.** (**A**, **B**) The visualization between the methylation level and the RP2 expression. (**C**, **D**) The Kaplan–Meier survival of the promoter methylation of RP2. (**E**, **F**) The distribution landscape of methylation probes.

Although we were aware that RP2 was expressed significantly more frequently in gliomas and that this may result in a bad prognosis [19], we didn’t know what was causing it. DNA methylation affects glioma development by altering the expression of key genes [20]. Therefore, we will investigate RP2 related DNA methylation. In LGG, we found a negative correlation of methylation values of cg24511534, cg04586456 and other 7 methylation probes and RP2 expression levels (Figure 4A). In GBM, there was a negative association between the methylation values of the methylation probes cg00433220, cg00347850, and other 10 methylation probes and the expression levels of RP2 (Figure 4B). We further explored how the hypomethylation of these sites would affect the prognosis of glioma with MethSurv, the result of which showed that patients with lower RP2 methylation had worse OS than those with higher RP2 methylation (*P* < 0.05) (Figure 4C, 4D). Besides, we also visualized the distribution landscape of methylation probes for RP2 on the chromosome, with cg04586456, cg25366157 and other 8 methylation probes on island, cg22522912 and cg14091713 on N_ Shore, and cg00433220 on S_Shore (Figure 4E, 4F).

In summary, there was a negative association between the expression of RP2 and its methylation level. It can be concluded that High RP2 expression in gliomas is caused by diminished RP2 methylation, which ultimately results in a bad prognosis for glioma patients.

### RP2-associated functional enrichment pathway

**Figure 5:**
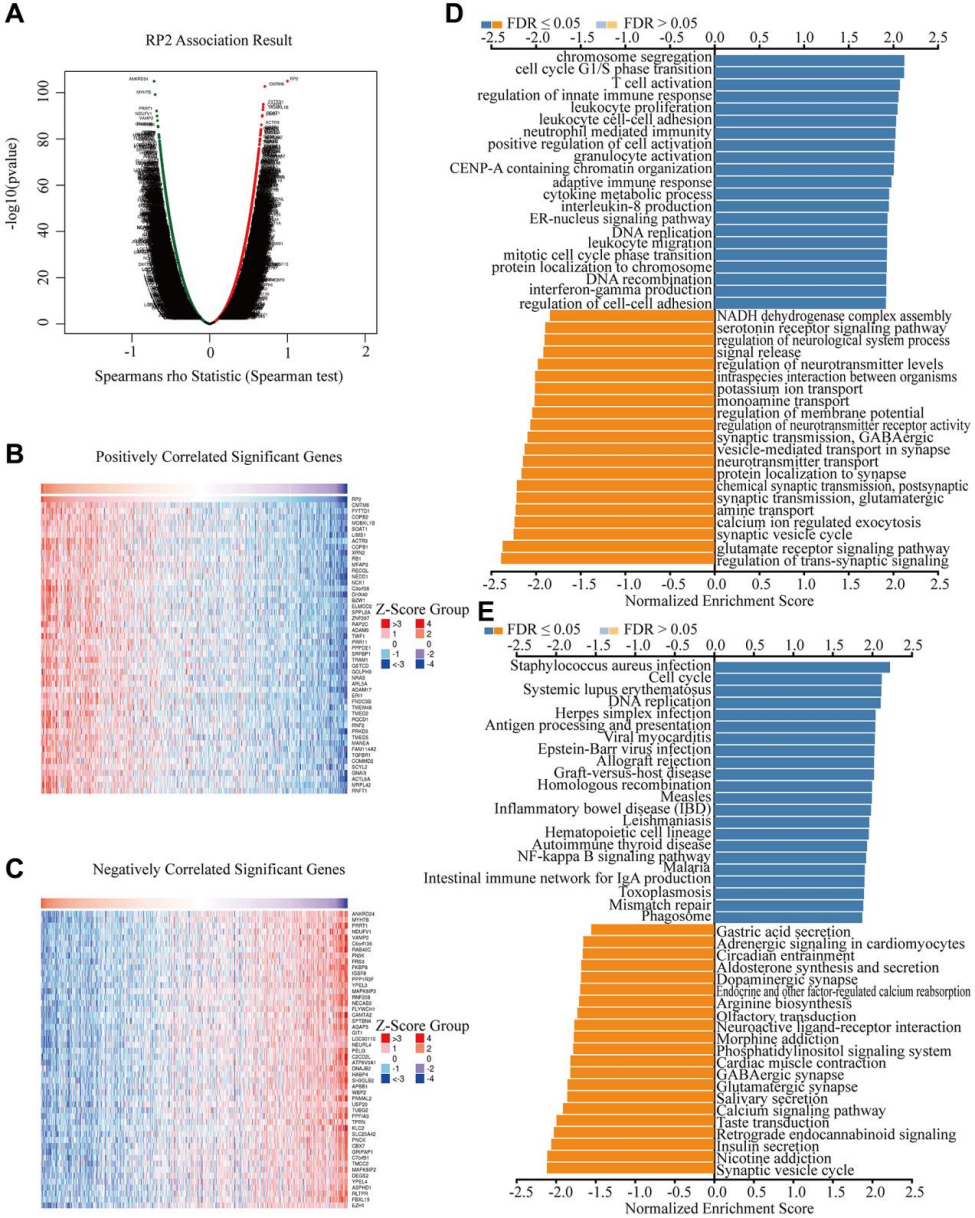
**Potential biological processes that RP2 involved in.** (**A**) Differential gene expression in GBMLGG. (**B**, **C**) The heat map respectively displayed the top 50 positively correlated and negatively correlated genes of RP2 in GBMLGG. (**D**, **E**) GO and KEGG enrichment analysis in GBMLGG of RP2.

**Figure 6:**
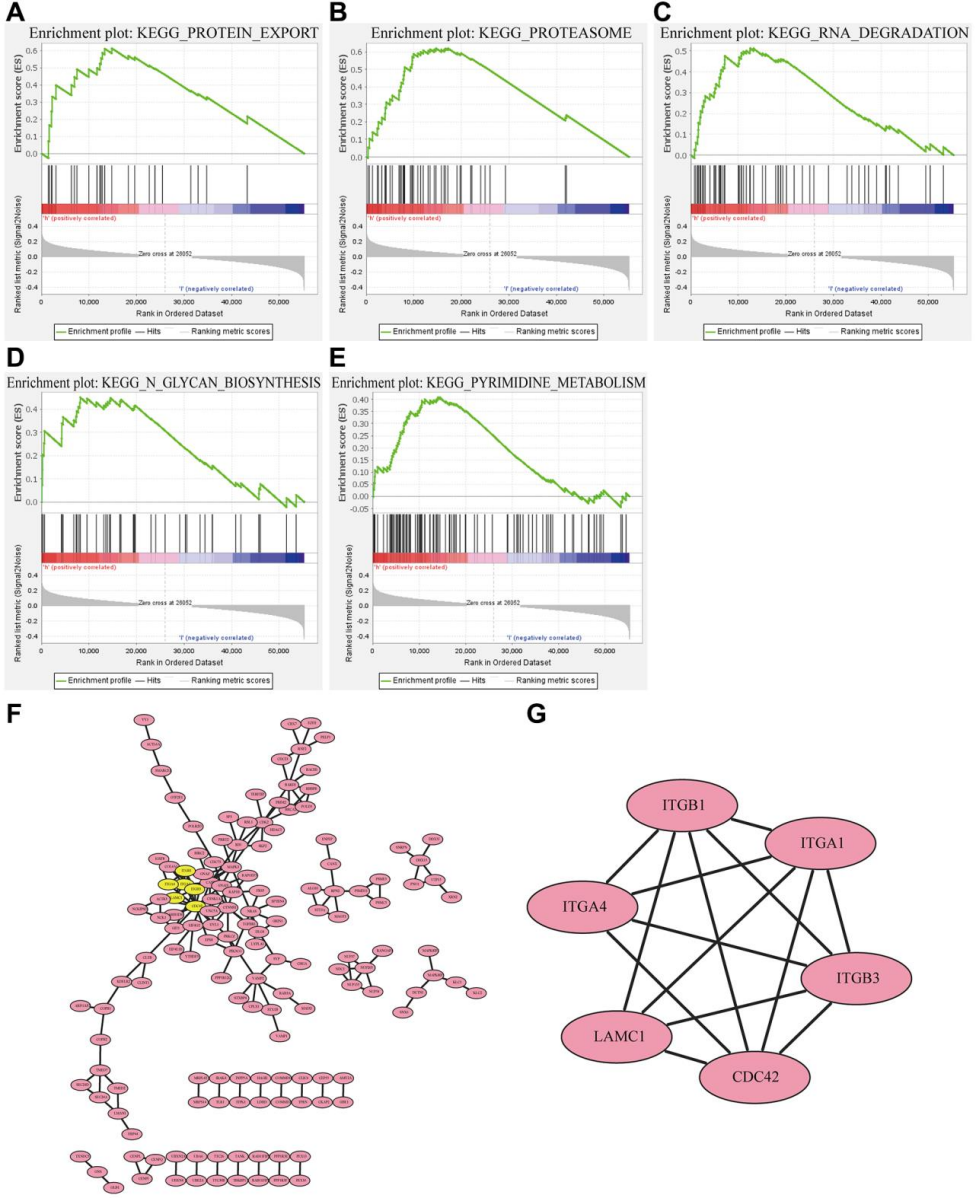
RP2-associated (**A**–**E**) pathways and (**F**) protein interaction networks as well as (**G**) the most correlated clusters.

Since RP2 presents a clear value in terms of patient prognosis, we produced a landscape of RP2-related genes to further investigate its functional regulatory pathways. The results came out that among the 20119 correlated genes identified, 7081 genes were positively associated (red dots) and 6743 genes were negatively associated (dark green dots) with RP2 expression (FDR <0.01) (Figure 5A). Therefore, we individually took out the top 50 positively and negatively linked genes to make a correlation heat map (Figure 5B, 5C). Gene ontology (GO) showed that RP2 may be involved in processes related to T cell activation, cell cycle, G1/S phase transition, and interferon production (Figure 5D). Kyoto Encyclopedia of Genes and Genomes (KEGG) pathway analysis displayed that RP2 was positively associated with processes such as antigen processing presentation and cell cycle (Figure 5E). Additionally, GSEA displayed that RP2 was closely related to protein export, proteasome, and RNA degradation, and so on (Figure 6A–6E). These findings suggested that RP2 may be engaged in processes related to cell cycle and organismal immunity. To further understand the mechanism of RP2 regulatory function, we selected genes with RP2 correlation located in the top 500 to produce clusters of RP2-related protein interactions. The clusters of proteins most associated with RP2 are shown by Figure 6F (yellow). We selected them to visualize them individually (Figure 6G), and the results showed that RP2 had protein interactions with ITGA4 and CDC42, among others, and verified that these proteins show correlation with immune response and metastatic spread of tumor cells, confirming our speculation about the function of RP2. We speculated that RP2 is associated with the cell cycle and immune infiltration.

### Structural and physical interactions of proteins

Proteins often need to dock several tertiary structures to each other to form quaternary structures for biological functions and metabolic reactions [21]. To investigate the potential mechanisms of protein physical interactions, we first selected RP2 to construct a network of proteins with which it can interact (Supplementary Figure 2A). The results showed that RP2 and proteins such as ARL3 and WDR83 can form strong physical interactions. The secondary structures of RP2, ARL3, and WDR83, as well as their possible chemical modifications, such as phosphorylation and ubiquitination were visualized (Supplementary Figure 2B–2D). Besides, the peptide chain encoded by RP2 coiled and folded with ARL3 and WDR83 form a quaternary structure as shown in Supplementary Figure 2E, 2F. We speculate that these two structures may be a form of RP2 involved in the regulation of cell cycle and immunity.

### RP2 expression is correlated with immune infiltration in glioma tissues

**Figure 7:**
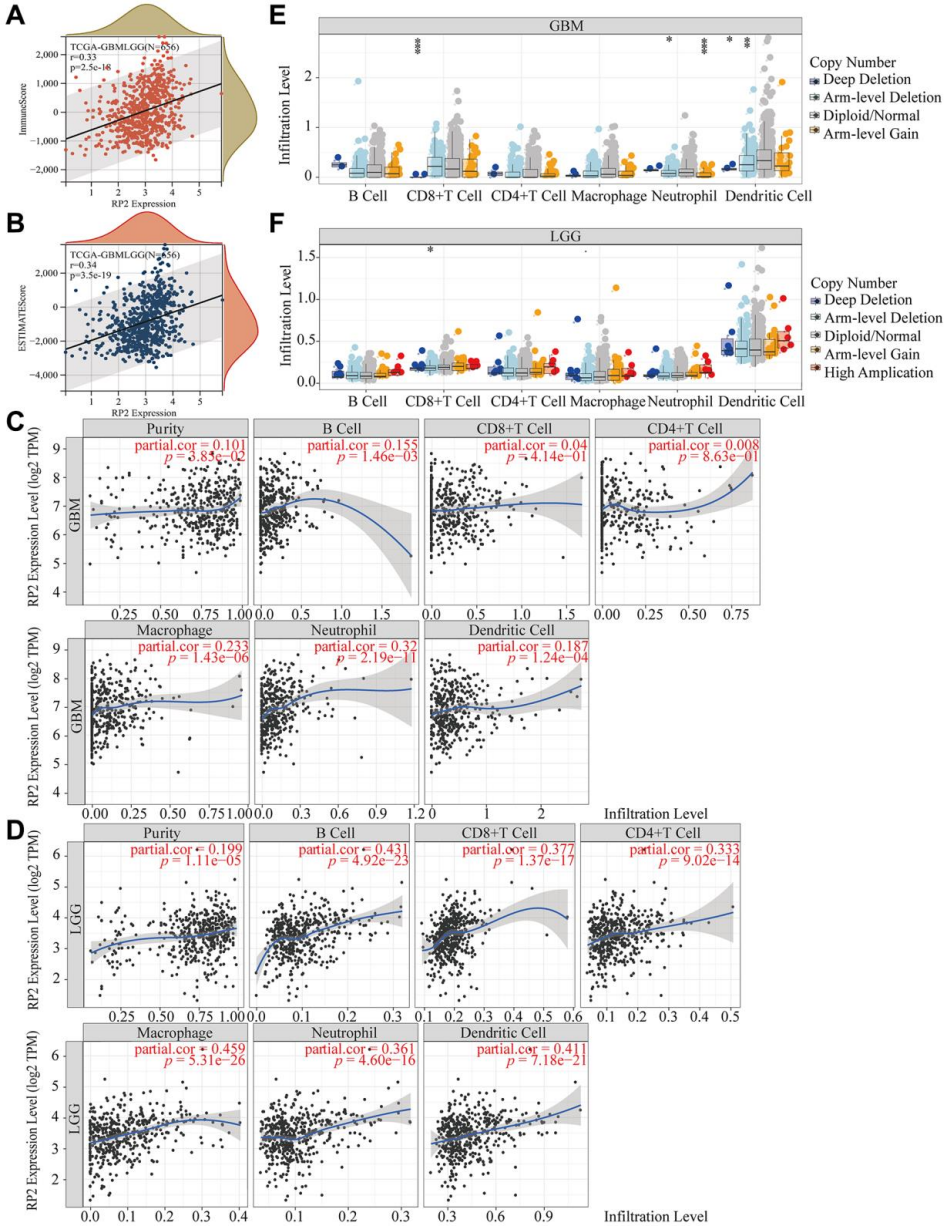
**RP2 is associated with tumor immune microenvironment in LGG and GBM.** (**A**, **B**) Correlation between RP2 and immune score and estimated score. (**C**, **D**) Correlation between tumor infiltrating immune cells and RP2 expression. (**E**, **F**) Correlation between CNV of RP2 and the degree of immune cell infiltration in LGG and GBM.

To investigate the part played by RP2 in influencing tumor immune microenvironment during tumor development, the Immune score (*P* = 2.5e-18, r = 0.33) and Estimate score (*P* = 3.5E-19, r = 0.34) of glioma samples have been analyzed. Both showed a clear correlation with RP2 expression (Figure 7A, 7B). Infiltration of six different types of immune cells were shown to be correlated with RP2 expression in glioma. The findings displayed that RP2 expression was definitely correlated with tumor purity in LGG and GBM, and showed an up-regulated relationship. In addition, we analyzed correlations between RP2 expression and multiple gene markers of immune cells in GBM and LGG (Tables 1, 2). As a result, we found RP2 was positively associated with the infiltration of Neutrophils, CD4+T cells, B cells, Dendritic cells, and Macrophages in glioma (Figure 7C, 7D). Since copy number variation (CNV) affects the degree of immune cell infiltration in gliomas [22], we looked into how copy number variation affects immune cell infiltration in LGG and GBM. Our results showed that high amplification notably promoted CD4+ T cell infiltration in LGG, however, in GBM, deep deletion, arm-level deletion and arm-level gain all reduced CD8+ T cell, neutrophil and dendritic cell infiltration to varying degrees (Figure 7E, 7F). In conclusion, our results showed an association between RP2 and tumor cell infiltration in glioma.

**Table 1:** Correlation analysis of RP2 with gene markers of different types of immune cells in GBM.

**Description**	**Gene markers**	**GBM**			
		**None**		**Purity**	
		**Cor**	***p*-value**	**Cor**	***p*-value**
**B cell**	CD19	−0.090419353	0.266339351	−0.081611178	0.343096609
	CD79A	0.024337451	0.765235249	0.073576658	0.392849699
**T cell (general)**	CD3D	−0.094950688	0.24301526	−0.001144837	0.98940664
	CD3E	−0.086157908	0.289277078	−0.001880643	0.982598966
	CD2	−0.026768124	0.742303068	0.086923807	0.312491425
**CD8+ T cell**	CD8A	0.027766609	0.733047762	0.077404037	0.3686316
	CD8B	−0.019694959	0.808856352	0.063096332	0.463871307
**Monocyte**	CD86	0.16528621	**0.041274574**	0.359953427	**1.56E-05**
	CSF1R	0.038176993	0.639055482	0.172026967	**0.044422116**
**TAM**	CCL2	0.008738424	0.914542108	0.113109156	0.188172112
	CD68	0.063758996	0.433212831	0.228203155	**0.007317004**
	IL10	0.020571164	0.80073866	0.180025202	**0.035285698**
**M1**	IRF5	−0.06442242	0.428434587	0.07994462	0.353074915
	PTGS2	0.146147454	0.071486262	0.224269972	**0.008423422**
**M2**	CD163	0.123738491	0.127449105	0.238567878	**0.004993773**
	VSIG4	0.125591385	0.12181493	0.311147239	**0.00021491**
	MS4A4A	0.143858979	0.076075412	0.327287916	**9.47E-05**
**Neutrophils**	CEACAM8	−0.048071507	0.555139227	−0.088884232	0.301661565
	ITGAM	0.009234316	0.909711622	0.13203481	0.124046351
	CCR7	0.079795076	0.326469493	0.171939882	**0.044531541**
**Natural killer cell**	KIR2DL1	−0.060250959	0.459406844	−0.032733417	0.704148747
	KIR2DL3	−0.204171782	**0.011358253**	−0.184354042	**0.031040173**
	KIR2DL4	−0.05070954	0.533613697	−0.062601865	0.467388003
	KIR3DL1	−0.121644174	0.134163332	−0.124808831	0.146181251
	KIR3DL2	−0.090673359	0.264992888	−0.083555575	0.331682395
	KIR3DL3	−0.09533997	0.241079987	−0.073218624	0.395162941
**Dendritic cell**	HLA-DPB1	0.031599721	0.697884216	0.167805498	**0.049991329**
	HLA-DQB1	0.126596572	0.118840339	0.204033154	**0.016780654**
	HLA-DRA	0.073345128	0.367182618	0.210153278	**0.013707693**
	HLA-DPA1	0.075613499	0.352519837	0.184992372	**0.030452392**
	CD1C	0.130238068	0.108584788	0.283530556	**0.00078678**
	NRP1	0.142793481	0.078292002	0.211580737	**0.013066048**
	ITGAX	−0.071187327	3.81E-01	0.020870859	8.09E-01

Bold value indicates *p*-values < 0.05.

**Table 2:** Correlation analysis of RP2 with gene markers of different types of immune cells in LGG.

**Description**	**Gene markers**	**LGG**			
		**None**		**Purity**	
		**Cor**	***p*-value**	**Cor**	***p*-value**
**B cell**	CD19	0.151886892	**0.000536286**	0.167434524	**0.000235936**
	CD79A	0.077067327	0.080292842	0.083721496	0.067423557
**T cell (general)**	CD3D	0.177874653	**4.84E-05**	0.229552378	**3.91E-07**
	CD3E	0.186633881	**1.98E-05**	0.227573857	**4.94E-07**
	CD2	0.237797138	**4.57E-08**	0.27442394	**1.05E-09**
**CD8+ T cell**	CD8A	−0.021539371	0.625443246	0.057386561	0.210423486
	CD8B	0.000579921	0.989515032	0.066657509	0.14562765
**Monocyte**	CD86	0.316800179	**1.71E-13**	0.412813708	**4.28E-21**
	CSF1R	0.237370457	**4.84E-08**	0.340230953	**2.03E-14**
**TAM**	CCL2	0.161849437	**0.000222528**	0.188819729	**3.26E-05**
	CD68	0.335041646	**5.31E-15**	0.392430536	**4.77E-19**
	IL10	0.264132504	**1.10E-09**	0.301153302	**1.77E-11**
**M1**	IRF5	0.2140266	**9.25E-07**	0.307452655	**6.36E-12**
	PTGS2	0.038916097	0.377671837	0.081023883	0.076774661
**M2**	CD163	0.27763733	**1.38E-10**	0.274652309	**1.02E-09**
	VSIG4	0.293663888	**1.01E-11**	0.35192554	**2.21E-15**
	MS4A4A	0.329120718	**1.68E-14**	0.348584637	**4.20E-15**
**Neutrophils**	CEACAM8	0.020992456	0.634250433	0.003218873	0.944041782
	ITGAM	0.272448913	**3.11E-10**	0.382404366	**4.31E-18**
	CCR7	0.182880293	**2.92E-05**	0.206942113	**5.07E-06**
**Natural killer cell**	KIR2DL1	0.098570827	**0.025147857**	0.107527954	**0.01869423**
	KIR2DL3	0.062212078	0.15820727	0.08136311	0.075543763
	KIR2DL4	0.202981691	**3.35E-06**	0.215437993	**2.00E-06**
	KIR3DL1	0.058714717	0.182973108	0.063539606	0.165458709
	KIR3DL2	0.094267072	**0.032281099**	0.108426563	**0.017723668**
	KIR3DL3	−0.001209509	0.978134213	0.008571398	0.85173036
**Dendritic cell**	HLA-DPB1	0.211510827	**1.25E-06**	0.249897344	**3.07E-08**
	HLA-DQB1	0.164678863	**0.000171691**	0.192138822	**2.34E-05**
	HLA-DRA	0.29123131	**1.52E-11**	0.335926839	**4.50E-14**
	HLA-DPA1	0.279018153	**1.11E-10**	0.319752121	**7.99E-13**
	CD1C	0.221116942	**3.90E-07**	0.22655268	**5.57E-07**
	NRP1	0.441753138	**4.64E-26**	0.412266256	**4.88E-21**
	ITGAX	0.169488294	**1.09E-04**	0.243918473	**6.64E-08**

Bold value indicates *p*-values < 0.05.

### Infiltration of some immune subtypes under the condition of high RP2 expression

**Figure 8:**
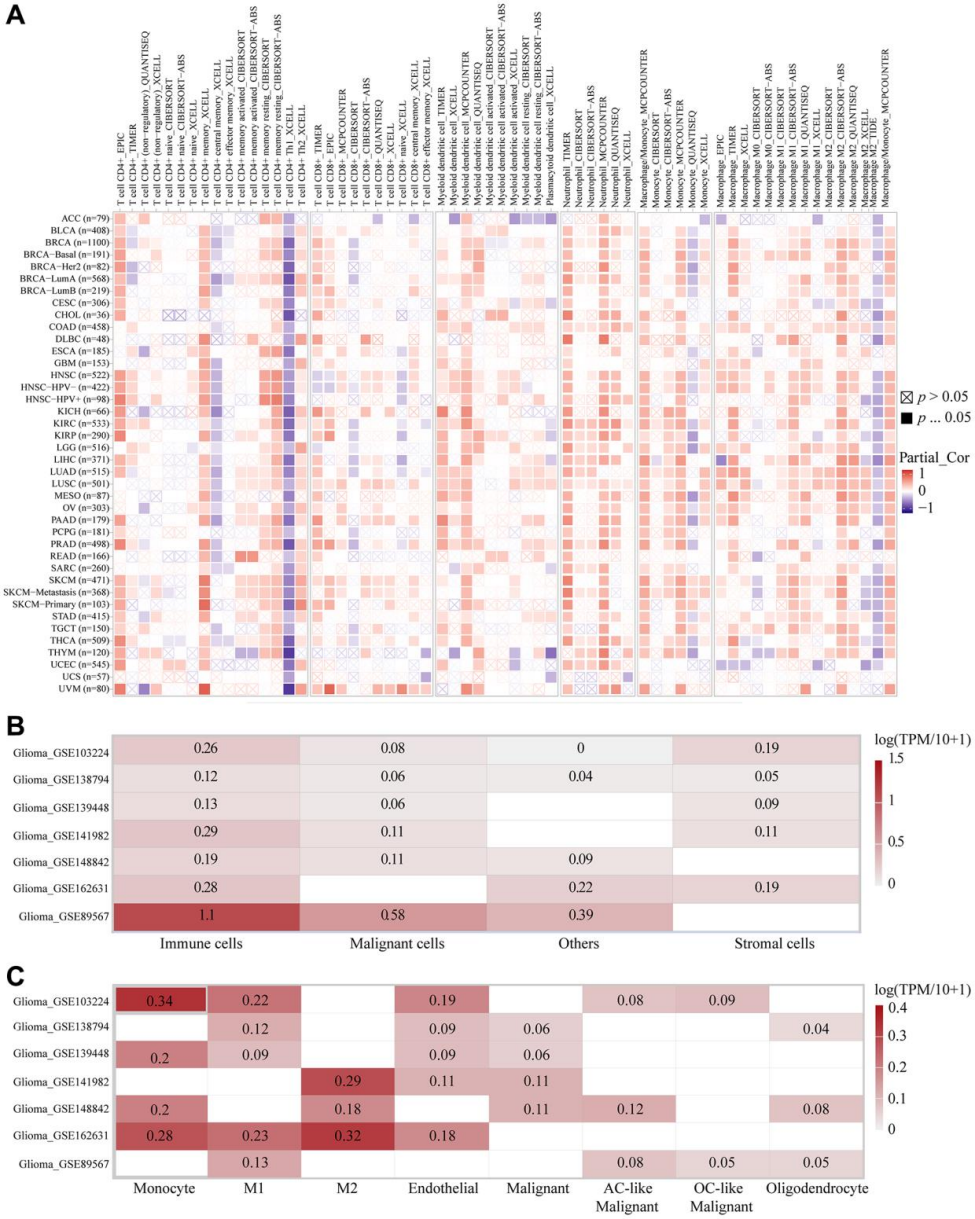
**The correlations of RP2 expression and the infiltration levels of immune cells.** (**A**) The correlations of RP2 expression and the infiltration levels of CD4+ T cells, CD8+ T cells, Myeloid dendritic cells, Monocyte, Macrophage and Neutrophil in cancers. Positive correlation in red and negative correlation in purple. (**B**, **C**) Single cell sequencing showed the expression of RP2 in different types of cells.

With the purpose of further exploring which immune cells the high expression of RP2 had the highest correlation with and the expression of RP2 in the TME, we performed the following analysis. The TIMER2.0 database was utilized to display the landscapes in which RP2 linked with diverse immune cell infiltrations in malignancies done on several quantitative immune infiltration platforms. The findings reveal that RP2 is associated with the immune infiltration of a wide spectrum of infiltrating cells, including CD4+ T cells, CD8+ T cells, Myeloid dendritic cells, Monocyte, Macrophage, and Neutrophil. Similarly, we found that in LGG and GBM, RP2 is negatively correlated with CD4+ T cells and positively correlated with Monocytes and Macrophages when highly expressed (Figure 8A). After that, to further investigate RP2 expression in the tumor microenvironment of gliomas, we carried out single-cell level analysis. We analysed seven single cell sequencing datasets, including glioma_GSE103224, through the TISCH website, and showed that RP2 is highly expressed in immune cells, particularly in M1 macrophages, M2 Macrophages, and Monocyte cells (Figure 8B, 8C). In conclusion, we discovered that immunosuppressive cell infiltration was positively connected with high RP2 expression in gliomas and was negatively correlated with immune helper cell infiltration. Furthermore, RP2 was significantly and relatively highly expressed in immunosuppressive cells, like M2 Macrophages, in TME.

### The dynamics of macrophage cells during glioma progression

**Figure 9:**
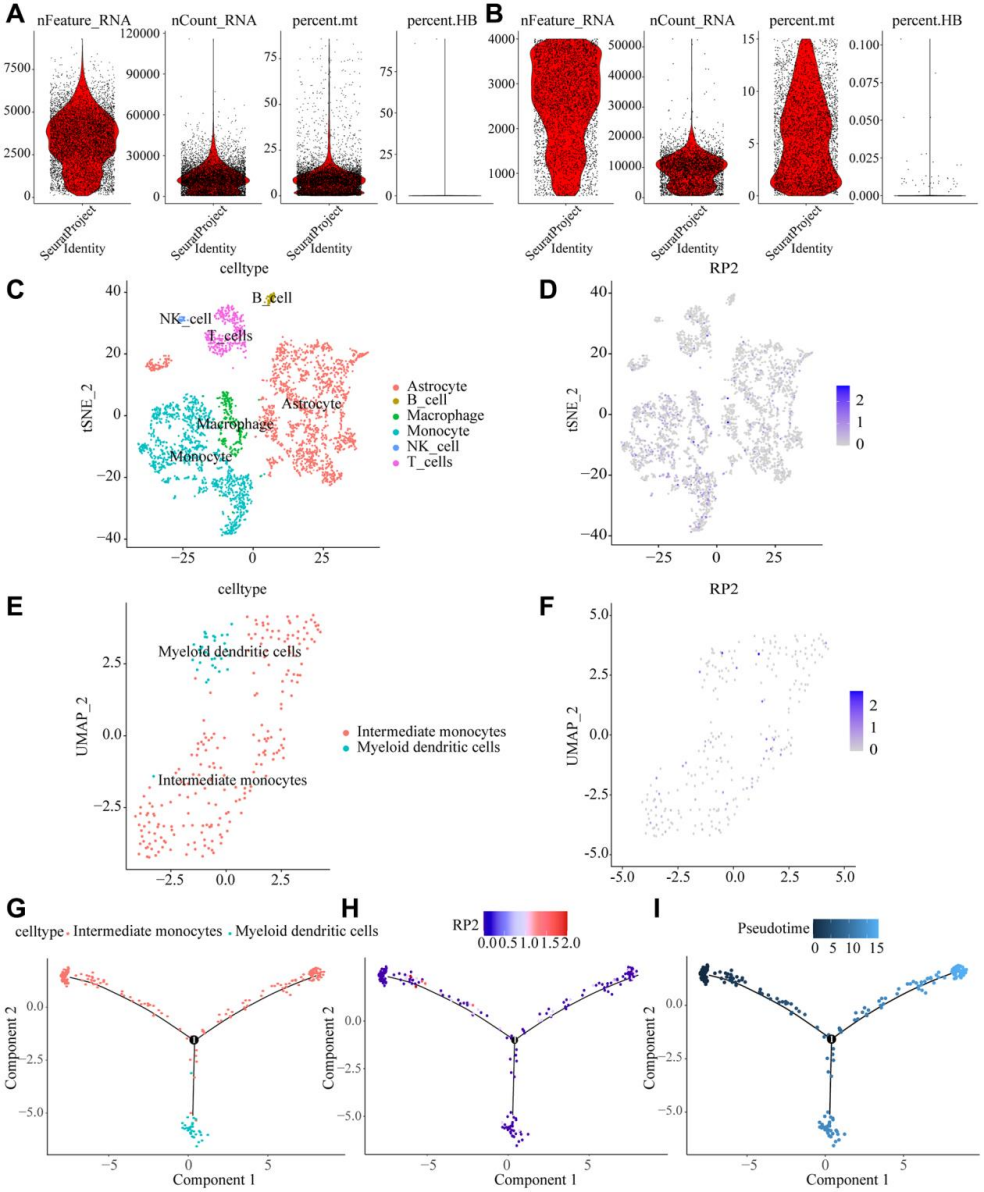
**Macrophage dynamics in glioma progression.** (**A**, **B**) Quality control removal of low-quality cells (**C**) tSNE cell cluster analysis differentiating cell types in the tumor microenvironment. (**D**) Tumor cells were divided into 13 clusters showing RP2 expression sites in the tumor microenvironment. (**E**) Cell reclustering was performed by UAMP method, and macrophage types were analyzed. (**F**) expression of RP2 was shown in the reclustering cells. (**G**) Evolutionary tracks of all kinds of cells after dimension reduction (**H**) expression of RP2 changes with pseudo time (**I**) differentiation tracks of macrophages in glioma, with cluster color code.

To describe the landscape of various cellular presence in the tumor microenvironment, we selected single-gene transcriptomes for clustering analysis, and after quality control to filter out irrelevant cells, Linear dimensionality reduction were used and visualization by t-distributed stochastic neighbor embedding (t-SNE) method. Cells from tumor and normal tissues were divided into 15 clusters (Figure 9A, 9B), and then clustered into six main categories according to the genetic characteristics of different subclusters: astrocytes, macrophages, monocytes, natural killer cells, T cells, and B cells, with monocytes and macrophages accounting for a large proportion of the tumor microenvironment. Meanwhile, we found that RP2 was mainly expressed in monocytes and macrophages (Figure 9C–9F).

We analyzed macrophages by downscaling, unsupervised clustering and trajectory analysis, and found that they could be further identified as Intermediate monocytes and Myeloid dendritic cells (Figure 9G), and the expression of RP2 did not show significant differences among these two types of cells (Figure 9H). The dynamic cellular states in the tumor microenvironment were visualized by our Monocle algorithm, and pseudo-temporal trajectory analysis showed that Intermediate monocytes tend to be depleted in a trajectory with tumor progression, while RP2 would show low expression in the early stages of cell development and higher expression over time, while expression was gradually downregulated after the node (Figure 9I). The above results suggested that the degree of RP2 gene expression influences the progression of the glioma tumor microenvironment and it might play an important part in the evolution of tumor microenvironment in glioma patients.

### Chemokines associated with glioma

**Figure 10:**
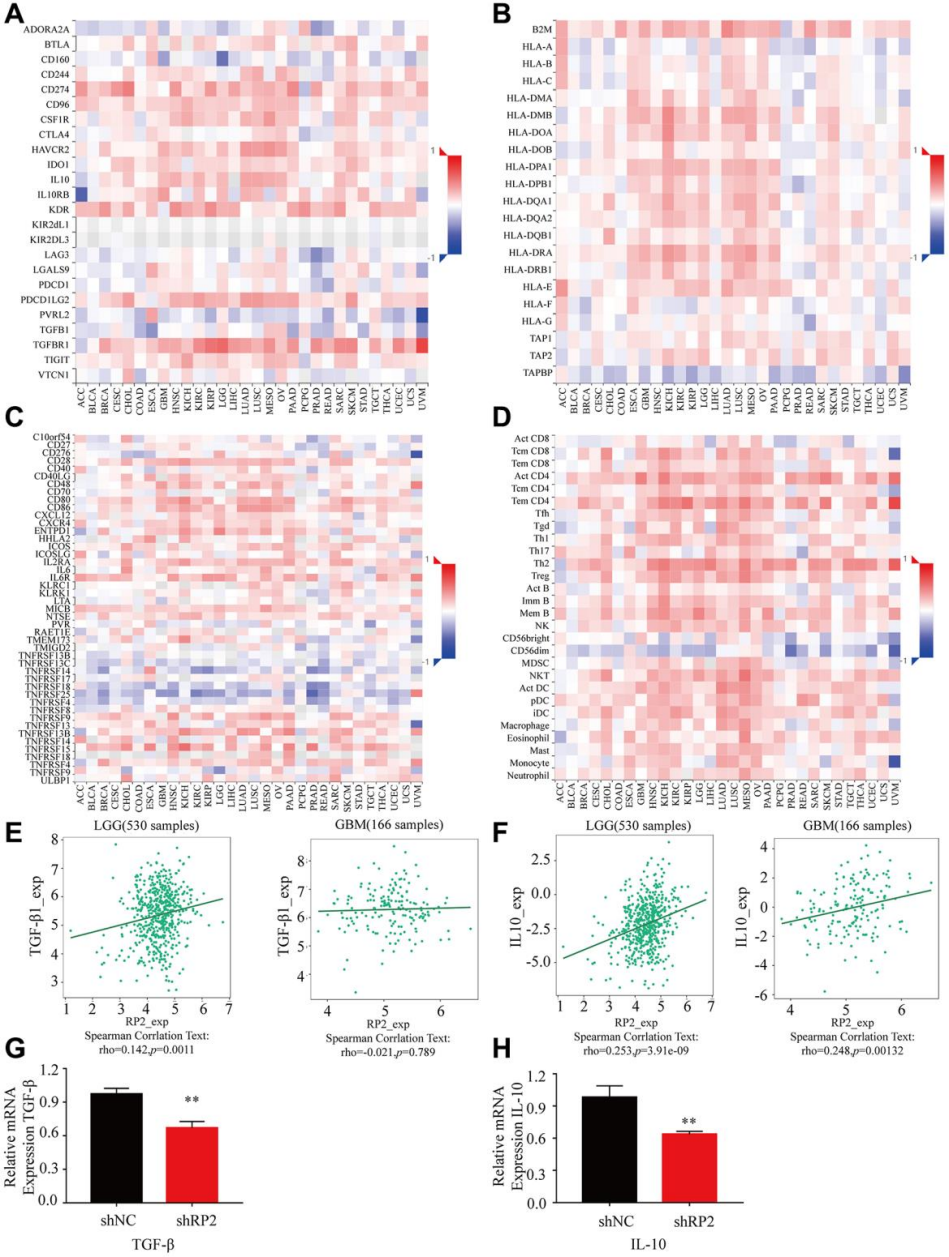
**The expression of various cytokines when RP2 is highly expressed in LGG and GBM.** (**A**) Immunoinhibitor. (**B**) MHC molecule. (**C**) Immunostimulator. (**D**) Lymphocyte. (**E**, **F**) RP2 was associated with the expression of TGF-β and IL-10. (**G**, **H**) RP2 was positively correlated with TGF-β and IL-10 mRNA expression.

Macrophages can secrete many cytokines, which play a key role in the tumor microenvironment and development of glioma. To investigate the key effect made by various cytokines produced by macrophages in TME, we visualized the contribution of different types of it in heat map form, and the results showed that: immune inhibitor factors such as TGFBR1 and IL10; MHC molecules such as B2M and HLA-A; immunostimulatory factors such as CD28 and CD48; lymphokines such as Act CD8 and Act CD4 had a positive correlation with RP2 in both GBM and LGG (Figure 10A–10D), Noteworthily, it is conformed that Macrophages may promote the progression of glioma through secrete cytokines like IL10 and TGF-β. We analyzed the correlation between RP2 and these two (Figure 10E, 10F), and found that they were positively correlated. In addition, we also verified them with experiments (Figure 10G, 10H). In conclusion, we speculated that macrophages may cause the poor prognosis of glioma by secreting these cytokines or influenced by them.

### Effect of over-expression RP2 on immunosuppression and immunotherapy of glioma

**Figure 11:**
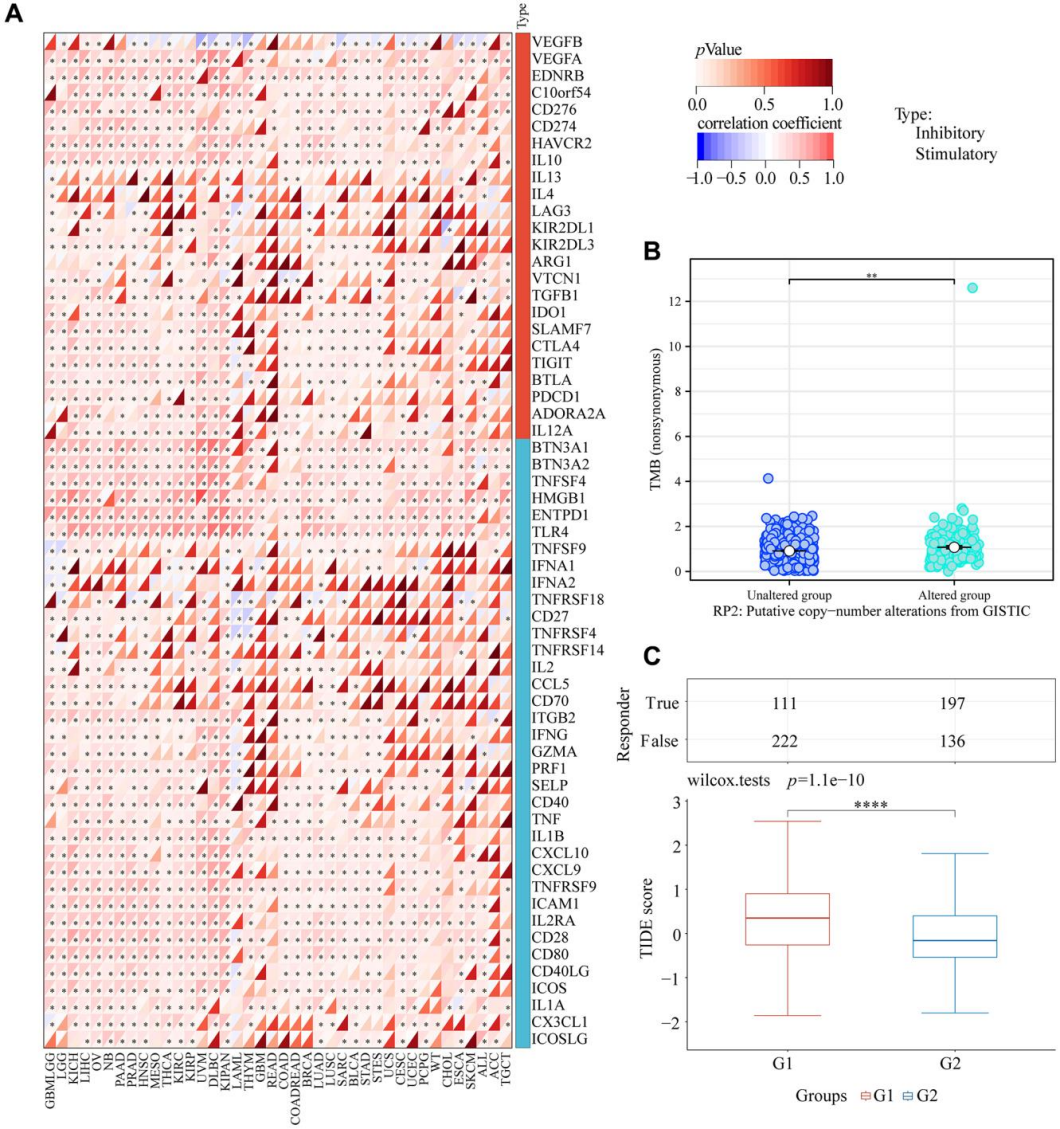
**RP2 and immunotherapy.** (**A**) Correlation between RP2 and various cell checkpoints. (**B**) TMB was low when RP2 was highly expressed. (**C**) TIDE score was high when RP2 expression was high which led to a poor immunotherapy effect.

In order to further investigate the impact of RP2 upon this immune escape microenvironment and immunotherapy in glioma, we also analyzed the correlation between RP2 and immune checkpoint and the associated TMB, and TIDE. The findings demonstrated a favorable correlation between the expression of most cell checkpoints and the expression of RP2 in GBMLGG, demonstrating that RP2 overexpression might disturb the normal cell cycle and promote the proliferation of glioma (Figure 11A). We also analyzed the relationship between the expression of some classic immune checkpoints and RP2 in glioma, and found that the expression of immune checkpoints increased when RP2 was high, indicating enhanced immune escape (Supplementary Figure 3). The expression of RP2 in GBMLGG was positively related with TMB, suggesting that high expression of RP2 may promote the development of cancer by affecting gene mutations in glioma, meanwhile, a low mutation rate is not conducive to immunotherapy (Figure 11B). Finally, TIDE score was analyzed and it was found that the treatment of the high RP2 group was not sensitive to immune checkpoint blockade (ICB) and the therapeutic effect was poor (Figure 11C). In conclusion, we known that high RP2 expression was conducive to immune escape of glioma, but not conducive to immunotherapy.

### Gene-drug interactions and drug sensitivity analysis

**Figure 12:**
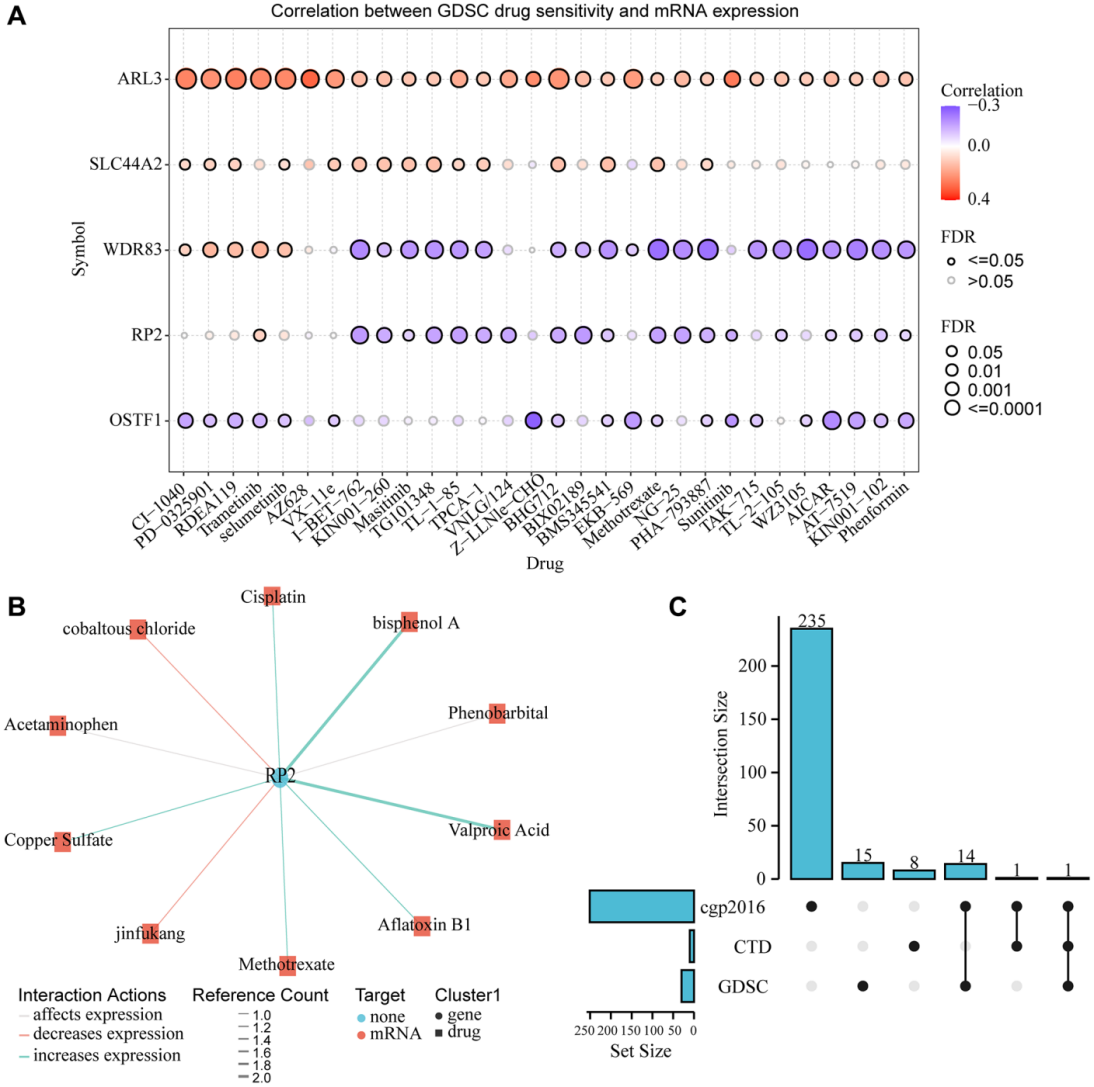
**Gene-drug interaction and drug sensitivity analysis.** (**A**) Relationship between RP2 expression and drug sensitivity in the GSCA database. (**B**) CTD showed the network of action between RP2 and drugs or small molecules. (**C**) Drug susceptibility results from three databases were displayed.

To explore the drug sensitivity of RP2 and genes highly associated with it, we conducted drug sensitivity analysis using the GDSC database, which showed that RP2 exhibited tolerance to 19 drugs or small molecules when highly expressed. And it showed sensitivity to Trametinib (Figure 12A). To explore the interaction between the pivotal gene and the available therapeutic agents for cancer, we used the CTD database to produce the interaction network between RP2 and drugs, and the results showed that cobaltous chloride and jinfukang were able to inhibit the gene expression of RP2, while six drugs or small molecules such as Methotrexate and Cisplatin were able to promote the expression of RP2 (Figure 12B). We compared three databases, cgp2016, CTD and GDSC, and concluded that Methotrexate was present in all three databases to regulate RP2 at the same time (Figure 12C). Patients are not sensitive to this drug when RP2 is highly expressed and it has been shown to increase RP2 expression, so this drug would not be the preferred option in treatment.

### The relationship between RP2 expression and m6A modification

In several cancer types, the effect of N6-methyladenosine (m6A) RNA modification has been proven [23]. By examining the TCGA-GBMLGG dataset, we explored the relationship among RP2 and the expression of 20 m6A-related genes in GBMLGG. RP2 expression was shown to be strongly and positively linked with 20 regulatory factors except FTO and IGF2BP2 (Supplementary Figure 4A, *P* < 0.05). We sought to find out if 20 distinct variables expressed differentially in glioma cells with high and low RP2 expression. The findings revealed that 20 variables expressed themselves more strongly in the “High RP2” group as compared to the “Low RP2” group (Supplementary Figure 4B). Meanwhile, based on RP2 expression levels, we separate samples into groups with high and low expression. M6A regulator networks depict a comprehensive picture of the correlation between the expression of m6A regulatory factors and the effect of regulatory factors on the prognosis of glioma. We found significant correlations in expression and prognosis not only between m6A regulatory factors of the same functional class, but also between writers, erasers and readers (Supplementary Figure 4C, 4D, Table 3). According to these findings, RP2 may be correlated with m6A modification in glioma, and the combined effect of METTL14, VIRMA, ALKBH5, YTHDC2 and METTL3 may eventually affect the progression and poor prognosis of glioma.

**Table 3:** Prognostic impact of m6A gene in the case of high or low expression of RP2.

**High RP2**	***p*-value**	**Low RP2**	***p*-value**
**IGF2BP3**	**4.42E-23**	**METTL14**	0.982063538
**IGF2BP2**	**3.02E-22**	**YTHDF3**	0.960606816
**FTO**	**3.08E-15**	**METTL3**	0.747635881
**YTHDC1**	**1.49E-14**	**RBM15B**	0.685309729
**WTAP**	**1.98E-14**	**YTHDC2**	0.637349611
**ZC3H13**	**3.67E-14**	**RBMX**	0.535317235
**YTHDF2**	**9.42E-11**	**VIRMA**	0.489728754
**METTL14**	**7.31E-08**	**HNRNPC**	0.425883752
**IGF2BP1**	**2.24E-05**	**RBM15**	0.114137498
**VIRMA**	**6.21E-05**	**ALKBH5**	0.072041983
**ALKBH5**	**0.002190185**	**IGF2BP1**	**0.045061783**
**YTHDC2**	**0.004426155**	**FTO**	**0.026184137**
**METTL3**	**0.006933739**	**YTHDC1**	**0.003886155**
**HNRNPC**	0.059620271	**HNRNPA2B1**	**0.002008863**
**RBM15**	0.174862774	**ZC3H13**	**0.001319473**
**RBMX**	0.202804513	**YTHDF1**	**0.000567207**
**HNRNPA2B1**	0.47095038	**YTHDF2**	**1.24E-05**
**RBM15B**	0.53264432	**WTAP**	**5.76E-07**
**YTHDF3**	0.6137567	**IGF2BP2**	**4.12E-19**
**YTHDF1**	0.664356478	**IGF2BP3**	**8.13E-22**

Bold value indicates *p*-values < 0.05.

## DISCUSSION

Glioma is the most frequent primary intracranial tumor of the central nervous system, with a high degree of malignancy, few diagnostic and therapeutic potions, a dismal prognosis, a propensity for recurrence and challenge in cure [24]. At present, there are many new directions for the treatment of glioma, such as electric field therapy, laser interstitial hyperthermia LITT technology and so on, and targeted therapy and molecular immunotherapy are the hot cutting-edge treatment technologies [25–27]. Some existing molecular biomarkers, such as GFAP, IDH1 and Ki-67 antigen [28–31], are helpful for the diagnosis of molecular subtypes, individualized treatment and clinical prognosis of glioma, but their sensitivity and accuracy are still lacking. Therefore, it is imperative to identify a more effective biomarker for glioma patients’ diagnosis, therapy, and prognosis assessment. In this study, a series of extensive and rigorous bioinformatic analyses and experimental validation were conducted to identify a new and powerful potential prognostic factor and therapeutic target for glioma.

In this study, we selected TCGA and GTEx datasets, combined with bioinformatics analysis, including differential gene analysis, Weighted correlation network analysis (WGCNA), multivariate analysis, survival analysis, and ROC analysis. Finally, RP2 was identified as a potential independent prognostic factor for glioma. One of the most crucial filtering steps in our research involved the flexible application of WGCNA, a systems biological approach to describe patterns of gene association between various samples. WGCNA is typically used to identify candidate biomarker genes or therapeutic targets based on association within gene sets and association between gene sets and phenotypes [9]. It enabled us to categorize genes into various modules and then choose the module that most closely correlated with the occurrence and development of glioma. After that, we could continue to use some other bioinformatics, like the survival analysis, multivariate analysis, and ROC, to further analyzed and finally screened RP2 as our research target, which is significant positive correlation with the occurrence and development of glioma.

We know that hypermethylation leading to silencing of gene expression has been shown to be a widespread tumor epigenetic phenomenon, and some evidence also suggest that it is reasonable and well documented that in certain settings, decreased the level of promoter methylation is directly associated with increased gene expression. For example, it has been reported that DNA hypomethylation may be the reason for the up-regulation of YTHDF2 in LGG, which increases the expression of YTHDF2 by regulating the transcription process of YTHDF2 and leads to poor prognosis of glioma patients. Another research reported that CD133 overexpression in BTSCs due to P2 hypomethylation underlies glioma recurrence. It is evident that not all genes are affected by methylation in the same way and that the contribution of epigenetics to transcriptional regulation may occur in a more complex and dynamic manner. In our study, RP2 expression was upregulated in glioma and the RP2 promoter was hypomethylated in glioma. Based on the current state of research, the following pathways may exist: under specific circumstances, promoter hypomethylation may provide mechanical blockage of DNA-repressor interactions, thereby promoting active gene transcription, or promoter hypomethylation can induce synergistic effects of distal regulatory elements to promote gene activation mechanisms.

Considering that RP2 is a potential prognostic factor of glioma, we were eager to know what biological processes does RP2 participate in glioma. We know that malignant cell proliferation is the main cause of glioma, and abnormal immune infiltration is also an important factor affecting tumorigenesis and development of glioma [32, 33]. It has been reported that FOXD2-AS1 can promote cell proliferation in glioma through regulating FOXD2-AS1/miR-31/CDK1 axis [34]. In addition, glioma cells express immunosuppressive cell surface molecules like HLA-G or release soluble immunosuppressive substances like TGF-β to inhibit anti-tumor immune responses and promote cancer progression [35]. And in our research, through the GO analysis, we found that RP2 is closely correlated with the biological processes related to cell proliferation, such as G1/S phase transition of cell cycle, DNA replication, mitotic cell cycle phase transition, chromosome protein localization, as well as the biological processes related to immune responses, such as T cell activation, leukocytosis, leukocyte-cell adhesion, and granulocyte activation. KEGG enrichment analysis also showed that RP2 is enriched in pathways related to cell cycle, DNA replication, antigen processing and presentation, and the occurrence and development of many autoimmune diseases and various types of inflammation. Besides, our corresponding experimental results also demonstrated that overexpression of RP2 contributes to cell proliferation in glioma. Based on the above information, it could be speculated that RP2 could promote the progression of glioma through influencing cell proliferation and immune infiltration.

Functional analysis of RP2-related genes also confirmed our speculation about the functions that RP2 involved in. In our study, we identified ARL3 and WDR83 as the most associated with RP2 in physical interaction, analyzed their secondary structures respectively, and visualized their binding to RP2 with vivid images. By checking the background information, we found that ARL3 can directly bind to RP2, and RP2 can act as the GTP-enzyme activating protein of ARL3, thus affecting intracellular microtubule regulation and protein transport, which can cause diseases such as retinitis pigmentosa [36–38]. At the same time, another article have confirmed that ARL3 is a prognostic biomarker for glioma, and its low expression predicts poor prognosis. RP2 can induce the hydrolysis of GTP ARL3. Therefore, we speculated that overexpression of RP2 may also affect the progression of glioma through the interaction with ARL3. As for WDR83, it is closely related to extracellular signal-regulated kinase (ERK) that is able to regulate many signal transduction pathways like Ras-Raf-MAPK signaling pathway [39]. And it has been reported that Ras can induce nuclear transcription by regulating the Raf-MEK-MAPK signaling pathways, thus promoting the cell proliferation of glioma [40, 41]. Based on the above, we conjectured that RP2 may also affect the progression of glioma via affecting these signaling pathways through WDR83. Physical interactions between proteins can often lead to connections in biological functions. So, the exploration of RP2 interacting proteins provides us a new angle and a new proof to study the specific mechanism of glioma, even if the precise process behind this is yet unknown, we will clarify it in further research.

Glioma is a rare immunologically cold tumor. Due to the specificity of its site, immunosuppression and anti-inflammation mechanisms suppress and counteract immune activity physiologically to limit the damage in brain tissue caused by immune responses [34]. Glioma utilizes the blood-brain barrier and the unique immunosuppressive microenvironment, resulting in a lack of tumor-infiltrating lymphocytes (TILs) and a relatively large number of immunosuppressive cells, which will lead to a poor immunotherapeutic outcome. According to reports, the immune infiltration in the TME is closely connected with the high expression of RP2. For example, the high expression of RP2 in KIRC is positively associated with the infiltration of a variety of immune cells, while it is highly negatively correlated with some immunosuppressive cells such as CD56brightCD16-NK and Treg cells [42]. In this paper, we found that the high expression of RP2 was positively correlated with many immunosuppressive cells, such as M2 Macrophages and Dendritic cells. What’s more, we also observed that RP2 was especially highly expressed in Monocytes and M2 Macrophages in the TME. Considering the immunosuppressive microenvironment in glioma, we chose Macrophages, which are rich in immunosuppressive background, for further study. It has been reported that tumor-associated macrophages (TAMs) are closely related to treatment failure and poor prognosis of glioma patients [6]. There is an article confirms that DHX9 can increase TAM infiltration in glioma by regulating the TCF12/CSF1 axis, and then promote its polarization into M2 Macrophage, which finally promote the progression of GBM [43]. And according to recent studies, glioma can be treated by obstructing the recruitment of Macrophages in TME, reducing the activity of TAMs or reshaping the phenotype of TAMs from M2 to M1 [44]. Other reports have also proved that in glioma, M2 Macrophages promote angiogenesis, while M1 Macrophages inhibit angiogenesis. Therefore, it is a good idea to treat glioma with anti-angiogenic therapy through regulation of TME [45]. So, combined with our results, we can speculate that high RP2 expression may promote the progression of glioma by influencing the infiltration and polarization of Macrophages.

Some studies have shown that CD8+ T cells can kill tumor cells, and TAM will help tumor cells to participate in immune escape. In our study, we found that when RP2 was highly expressed, CD8+T cell infiltration was slightly inhibited in LGG, but the degree of infiltration did not change significantly in GBM. Therefore, we speculated that CD8+T cells could not play its role in killing tumor cells when RP2 was highly expressed. Meanwhile, the degree of TAM infiltration was also significantly increased when RP2 was highly expressed. Besides, according to Figure8B and C, we could know that RP2 is mainly highly expressed in M2 Macrophages, and Figure9 also proved this result. So, we can speculate the immune escape effect supported by TAM is stronger than the killing effect to tumor cell of CD8+T cell in gliomas.

By modulating immunological response, cell proliferation, and other processes, cytokines also play significant and essential roles in the development of glioma [46, 47]. Meanwhile, it is also known that Macrophages can secrete a variety of cytokines to promote the development of glioma [48, 49]. So, we explored the expression of cytokines, especially cytokines secreted by Macrophages, at high RP2 expression. According to reports, M2 Macrophages in glioma can directly suppress the immune response by secreting IL-10, TGF-β, and other immunosuppressive molecules [50]. And our results did confirm that the high expression of RP2 is positively associated with IL-10, TGF-β in glioma. Therefore, we hypothesized that high RP2 expression could cause poor prognosis in glioma by affecting the infiltration of Macrophages and the secretion of IL-10 and TGF-β by Macrophages. At the same time, we think that this provides a clear direction for us to study the precise targeted Immunotherapy of glioma [51]. It has been proved that modulating the chemokine/chemokine receptor axis has also become a new therapeutic direction for glioma. A pre-clinical study showed that fused cytokines and antibodies could work together to motivate the immune system to attack tumors more strongly, leading to a qualitative leap in effectiveness [50, 52]. In addition, the “immune score” predicts the interaction of immune and non-immune factors and has been shown to be an independent marker of therapeutic efficacy before and after immunotherapy (PD-1/PD-L1, CTLA4) [53, 54]. Simultaneously, the high expression of immune checkpoints in RP2 overexpressed cancer tissues predicts a strong immune evasion ability of tumor tissues compared to normal cells. Nowadays, it has been demonstrated that CD5 protein on dendritic cells largely determines individual differences in the effect of immunotherapy [55], meanwhile, the effect of immune checkpoint blockade therapy often accompanied with a complex combination of molecular mechanisms. In brief, our results indicated the inapplicability of immunotherapy in the presence of RP2 overexpression and are informative for exploring the molecular mechanisms and clinical treatments associated with RP2. Moreover, our TIDE results also displayed that the effect of glioma immunotherapy was awful in the case of high RP2 expression, which just confirmed the above theory, and also suggested us that effective inhibition of RP2 overexpression in glioma is likely to improve the effect of immunotherapy and increase the survival hope of patients.

To the best of our knowledge, this is the first bioinformatics research of RP2 in glioma. In this paper, we identified a brand-new independent prognostic factor for glioma that is strongly immuno-related, and provides a new direction for the diagnosis and treatment of glioma in the future.

## Supplementary Materials

Supplementary Figures
