# Supplementary material for: Identified RP2 as a prognostic biomarker for glioma, facilitating glioma pathogenesis mainly via regulating tumor immunity
Source: Aging (Albany NY). 2023 Aug 18;15(16):8155–84. doi: 10.18632/aging.204962 (PMC10497014; doi:10.18632/aging.204962)
Supplement: Supplementary Figures [file aging-15-204962-s001.pdf]

SUPPLEMENTARY FIGURES

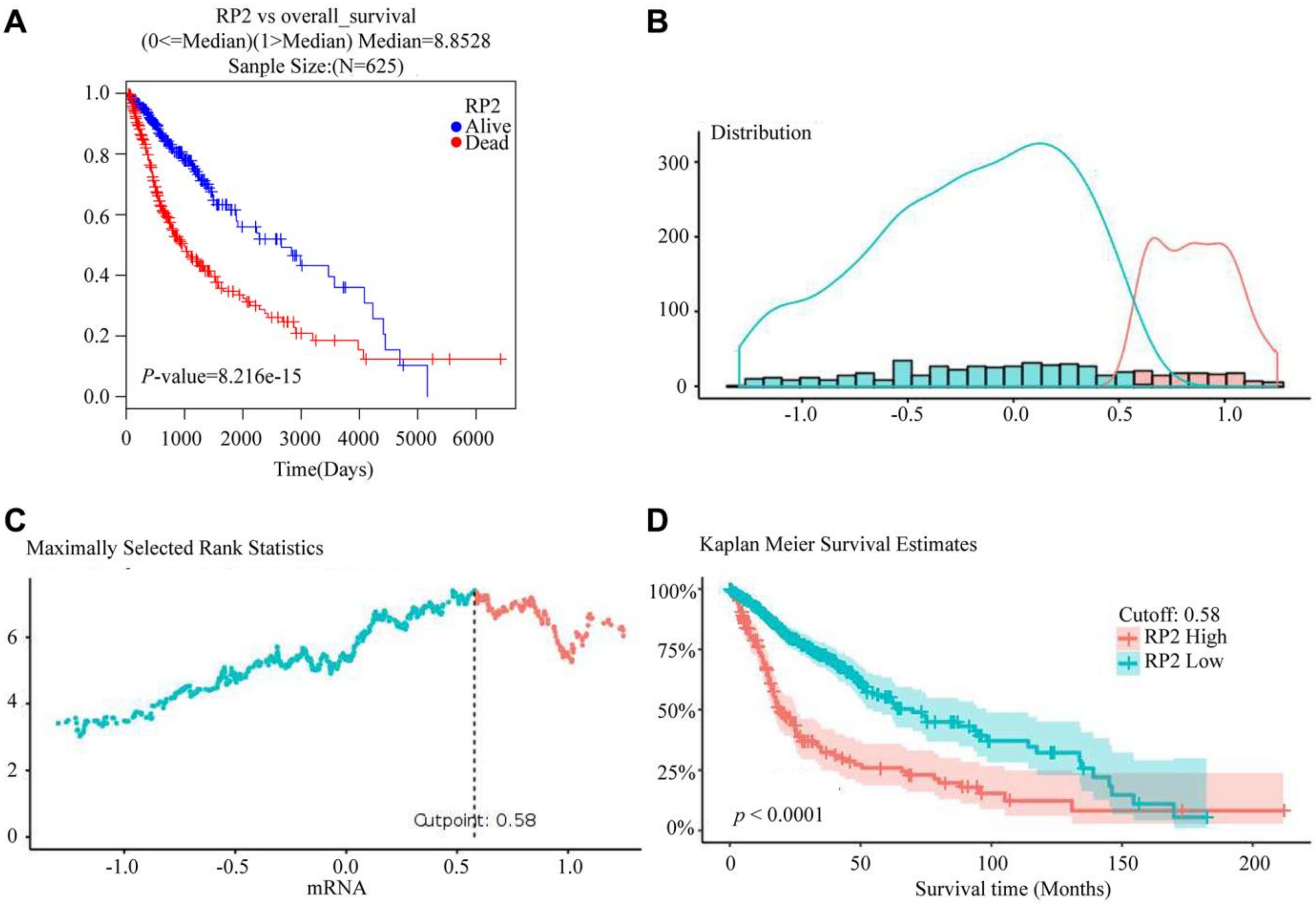

Supplementary Figure 1. Survival analysis validation from the other web sites (**A**) Overall survival was also examined using LinkedOmics. (**B**–**D**) Survival analysis examined via GlioVis.

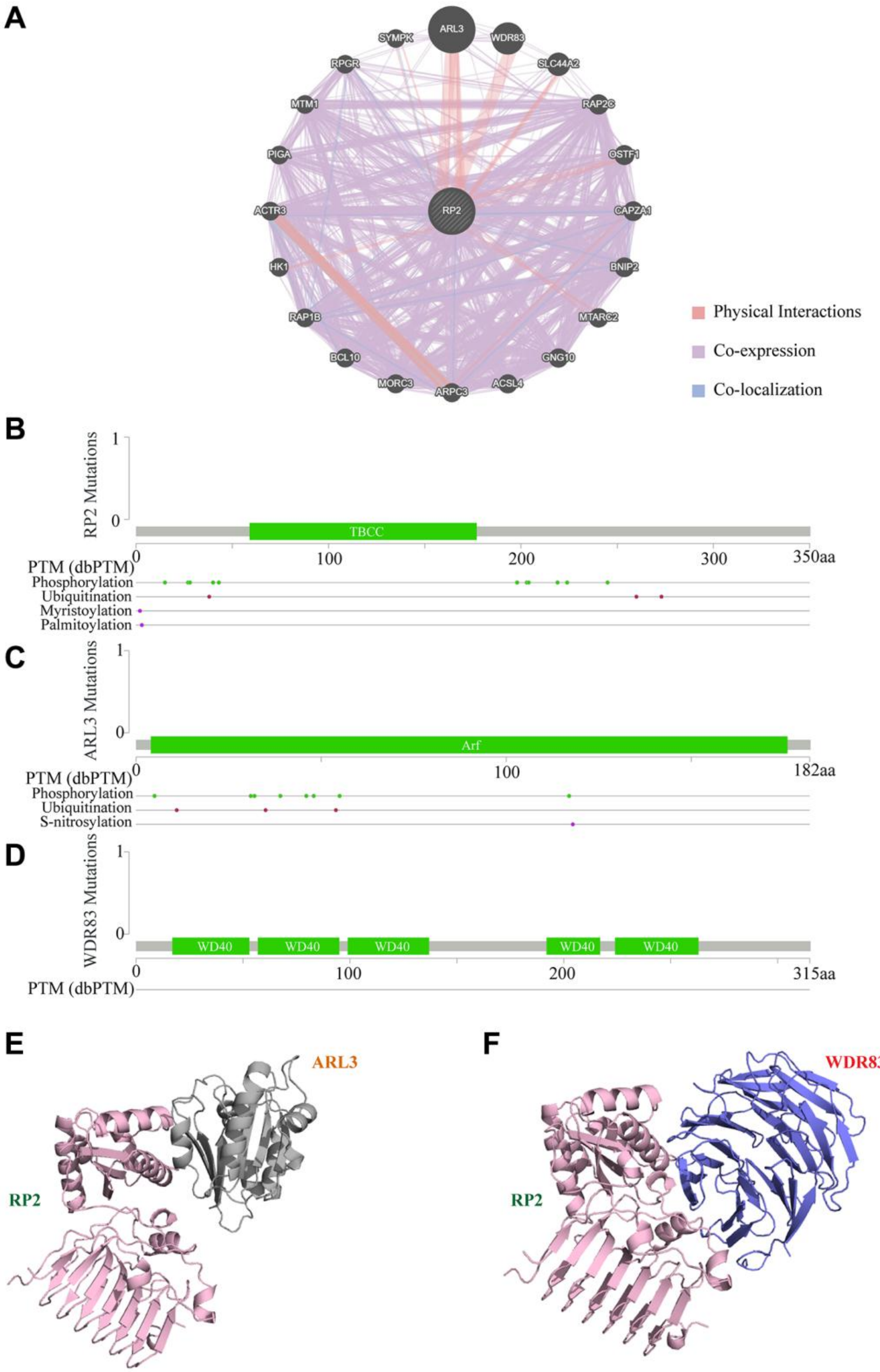

Supplementary Figure 2. Structural and physical interactions of RP2. (**A**) Physical interacting proteins of RP2. (**B**–**D**) Secondary structure and mutation sites of RP2, ARL3 and WDR83. (**E**, **F**) Structure diagram of physical interaction between RP2 and ARL3 and RP2 and WDR83.

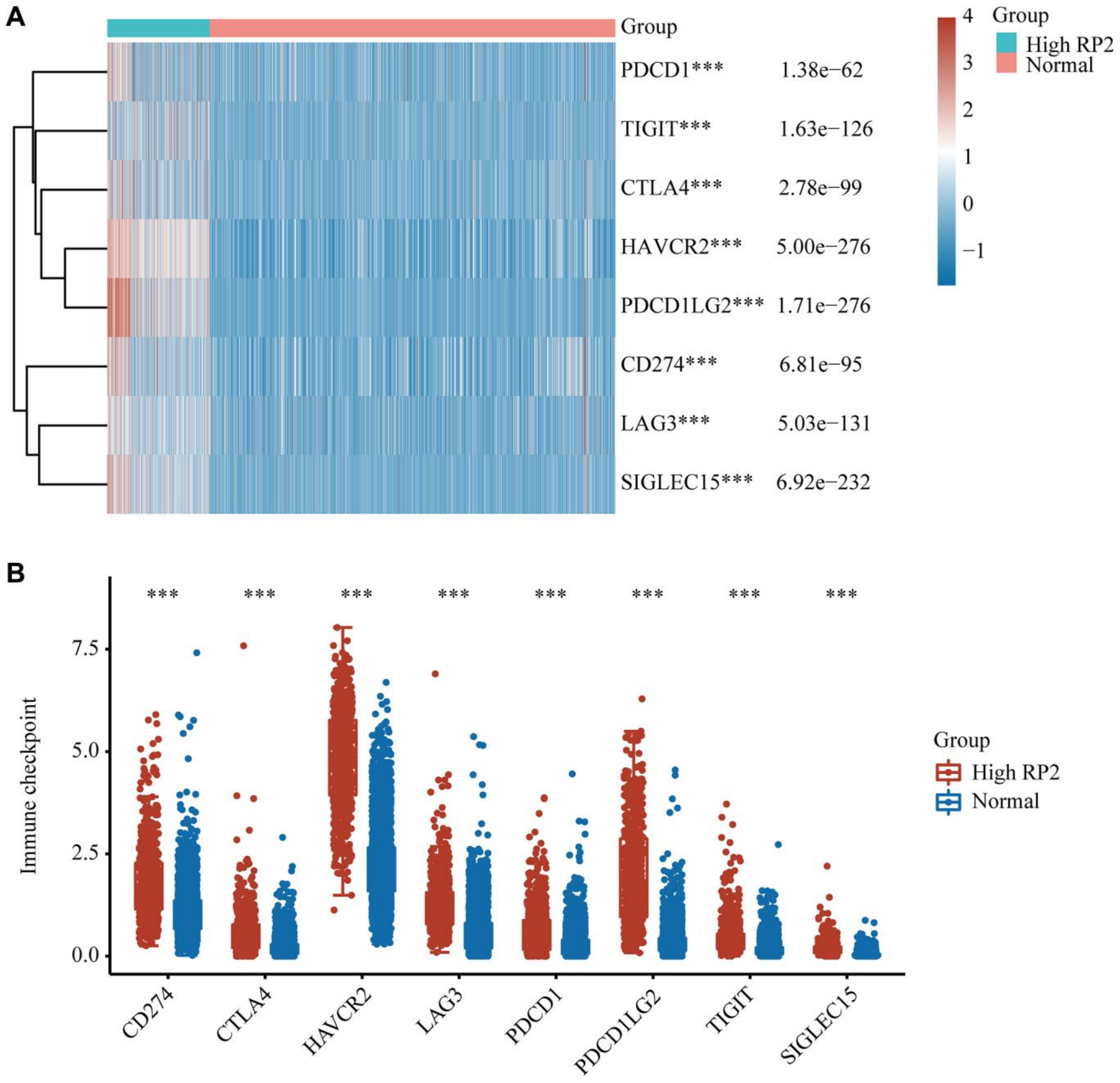

Supplementary Figure 3. Analysis of the correlation of some classical immune checkpoints and RP2 in expression. (**A**) Heatmap, (**B**) box diagram.

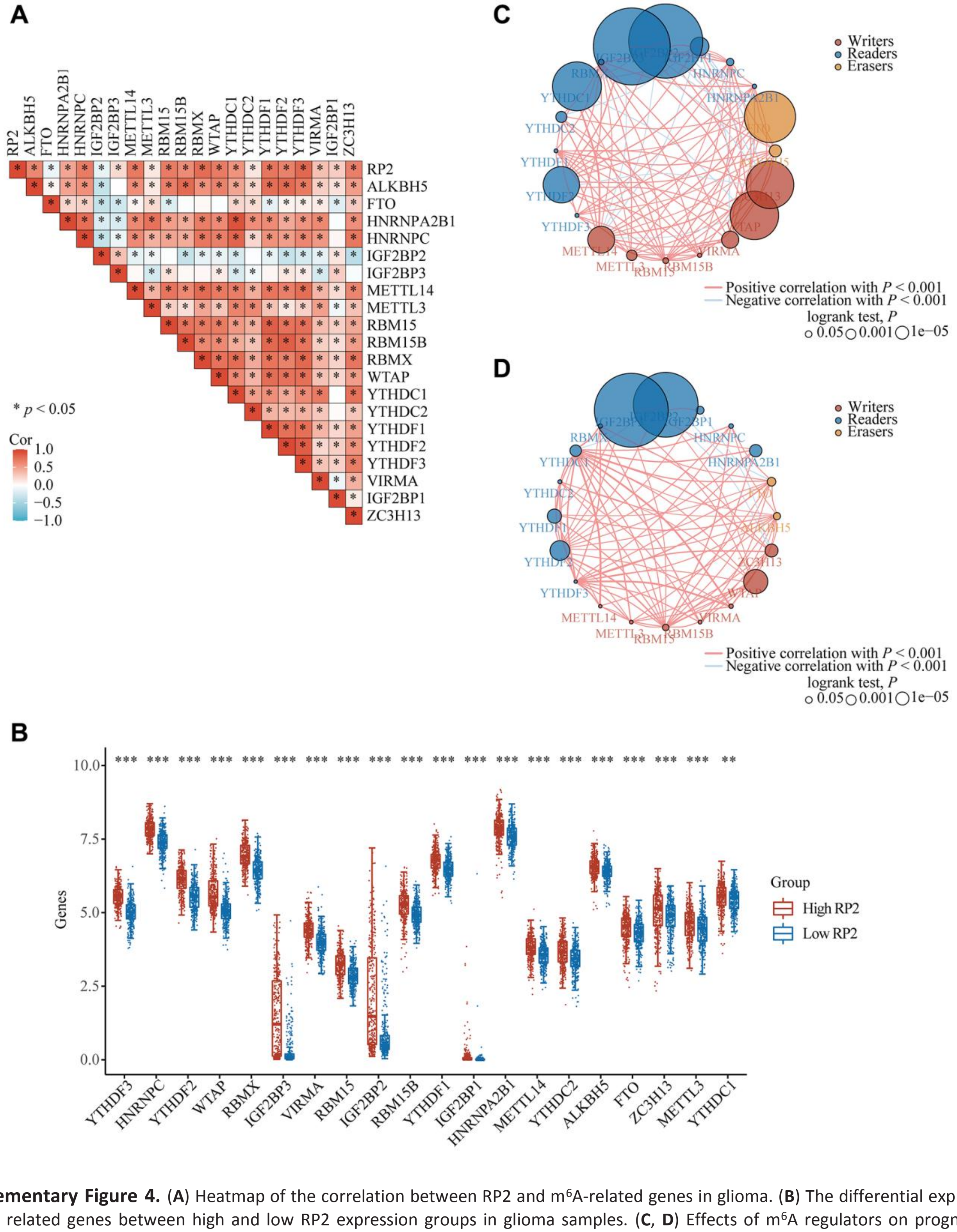

Supplementary Figure 4. (**A**) Heatmap of the correlation between RP2 and m^{6}A-related genes in glioma. (**B**) The differential expression of m^{6}A related genes between high and low RP2 expression groups in glioma samples. (**C**, **D**) Effects of m^{6}A regulators on prognosis of glioma with different expression of RP2.
